# Characterization of Novel Synthetic Polyphenols: Validation of Antioxidant and Vasculoprotective Activities

**DOI:** 10.3390/antiox9090787

**Published:** 2020-08-25

**Authors:** María Jesús Pérez de Vega, Silvia Moreno-Fernández, Gloria María Pontes-Quero, María González-Amor, Blanca Vázquez-Lasa, Beatriz Sabater-Muñoz, Ana M. Briones, María R. Aguilar, Marta Miguel, Rosario González-Muñiz

**Affiliations:** 1 Instituto de Química Médica, IQM-CSIC, Juan de la Cierva 3, 28006 Madrid, Spain; pdevega@iqm.csic.es; 2 Instituto de Investigación en Ciencias de la Alimentación (CSIC-UAM, CEI+UAM), C/Nicolás Cabrera 9, 28049 Madrid, Spain; silvia.moreno@csic.es (S.M.-F.); marta.miguel@csic.es (M.M.); 3 Instituto de Ciencia y Tecnología de Polímeros, ICTP-CSIC, Juan de la Cierva 3, 28006 Madrid, Spain; pontesquerogm@gmail.com (G.M.P.-Q.); bvazquez@ictp.csic.es (B.V.-L.); mraguilar@ictp.csic.es (M.R.A.); 4 Alodia Farmacéutica SL, Santiago Grisolía 2 D130/L145, 28760 Madrid, Spain; 5 Facultad de Medicina, Departamento de Farmacología, Universidad Autónoma de Madrid, Instituto de Investigaciones Biomédicas Hospital La Paz, Arzobispo Morcillo 4, 28029 Madrid, Spain; maria.gonzalezamor@uam.es (M.G.-A.); ana.briones@uam.es (A.M.B.); 6 Centro de Investigación Biomédica en Red de Enfermedades Cardiovasculares (CIBERCV), 28029 Madrid Spain; 7 Networking Biomedical Research Centre in Bioengineering, Biomaterials and Nanomedicine, CIBER-BBN, 28029 Madrid, Spain; 8 Instituto de Biología Molecular y Celular de Plantas (IBMCP, CSIC-UPV), Ingeniero Fausto Elio, 46022 Valencia, Spain; b.sabater.munyoz@gmail.com

**Keywords:** antioxidants, ORAC, ABTS, DPPH, polyhydroxyphenyl amides, polyhydroxyphenyl ureas, NADPH oxidase, vasculoprotection, *Saccharomyces cerevisiae*

## Abstract

Antioxidant compounds, including polyphenols, have therapeutic effects because of their anti-inflammatory, antihypertensive, antithrombotic and antiproliferative properties. They play important roles in protecting the cardiovascular and neurological systems, by having preventive or protective effects against free radicals produced by either normal or pathological metabolism in such systems. For instance, resveratrol, a well-known potent antioxidant, has a counteracting effect on the excess of reactive oxygen species (ROS) and has a number of therapeutic benefits, like anti-inflammatory, anti-cancer and cardioprotective activities. Based on previous work from our group, and on the most frequent OH substitutions of natural polyphenols, we designed two series of synthetically accessible bis-polyhydroxyphenyl derivatives, separated by amide or urea linkers. These compounds exhibit high antioxidant ability (oxygen radical absorbance capacity (ORAC) assay) and interesting radical scavenging activity (RSA) values (2,2′-azinobis-(3-ethylbenzothiazoline-6-sulfonic acid) (ABTS) and α,α-diphenyl-β-picrylhydrazyl (DPPH) tests). Some of the best polyphenols were evaluated in two biological systems, endothelial cells (in vitro) and whole aorta (ex vivo), highly susceptible for the deleterious effects of prooxidants under different inflammatory conditions, showing protection against oxidative stress induced by inflammatory stimuli relevant in cardiovascular diseases, i.e., Angiotensin II and IL-1β. Selected compounds also showed strong in vivo antioxidant properties when evaluated in the model organism *Saccharomyces cerevisiae*.

## 1. Introduction

In recent years, antioxidant compounds have become very popular because of their multiple benefits for health. Nutraceuticals are bioactive ingredients of fruits and vegetables, or products elaborated with natural substances, able to modulate metabolic processes. Among nutraceuticals, there are many antioxidant substances, like polyphenols [1,2,3]. There is an important number of compounds, biosynthesized by the secondary metabolism of plants, known as phytochemicals, which can be extracted from plants and have interesting therapeutic properties in the treatment of infectious diseases, cancer, hypercholesterolemia and immunological disorders [1,4]. Antioxidant compounds, in particular, polyphenols, have beneficial effects for human health, including, among others, anti-inflammatory, antihypertensive, antithrombotic and antiproliferative properties. They play important roles in protecting the cardiovascular and neurological systems, by having preventive or protective effects against the free radicals produced by normal metabolism in such systems [5,6,7,8,9]. As an example, resveratrol, a well-known potent antioxidant, has a counteracting effect to the excess of radical oxygen species (ROS) that is translated in a number of biological activities of therapeutic relevance, like anti-inflammatory and anti-cancer activities, as well as protection against cardiovascular diseases [10,11]. Antioxidants also have applications as additives in food and cosmetics for maintaining the quality of products and for extending their half-life [12,13].

Previous work from our group led us to the preparation of some polyhydroxy diphenylpropanones, which in addition to their activity as α7 nicotinic acetyl choline receptor positive allosteric modulation (nAChR, PAM), showed interesting antioxidant properties [14,15]. The two phenyl rings in these compounds are separated by a propanone linker. Since this connector could be susceptible to the action of natural nucleophiles from biomacromolecules, possibly leading to covalent complexes, we explored its change by a triazolyl ring, which excludes the possibility of covalent bonds with the linker. The resulting small collection of triazolyl polyphenols allowed us to study the influence of the number and position of OH groups on the antioxidant properties [16]. Although we obtained excellent antioxidants, the yield in the preparation of methoxythriazolyl intermediates was very low in many cases, limiting their future applications. Our interest on new antioxidant structures moved us to further explore the linker chain joining the two phenolic rings, looking for easy, effective reactions. As a result, a small collection of amide and urea polyhydroxyphenyl derivatives **1**–**18** was prepared and characterized for their antioxidant properties, using different methods, oxygen radical absorbance capacity (ORAC), 2,2′-azinobis-(3-ethylbenzothiazoline-6-sulfonic acid) assay (ABTS), and the α,α-diphenyl-β-picrylhydrazyl (DPPH) free radical scavenging test. Compounds with high antioxidant ability and acceptable aqueous stability were selected for further studies. The potential biological activity as antioxidants was tested in two biological systems highly susceptible for the deleterious effects of prooxidants in different inflammatory conditions, endothelial cells and whole aorta. Selected compounds showed protection against oxidative stress induced by inflammatory stimuli important in cardiovascular diseases, i.e., Angiotensin II and Interleukin 1β (IL-1β). Compounds with different in vitro antioxidant profiles were chosen for studying the in vivo antioxidant properties in a model organism, *Saccharomyces cerevisiae*. All of them showed recovery of stressed yeast growth comparable to model antioxidants resveratrol and vitamin C.

## 2. Materials and Methods 

### 2.1. Chemistry

General information, as well as the preparation and characterization of methoxy (OMe)-substituted intermediates (**19** to **36**) is described in the Supplementary Material document. 

### 2.2. Preparation of Polyhydroxylated Amides (1 to 10)

General procedure for the deprotection of methoxy groups. To a previously cooled solution (0 °C) of the corresponding methoxy-substituted compound (1 equivalent) in dried CH_2_Cl_2_ (15 mL), a 1M solution of BBr_3_ in DCM (2 equivalents for each heteroatom) was slowly added under Ar atmosphere. After stirring 24–48 h at room temperature under Ar, monitoring the total disappearance of the methoxy groups by HPLC-MS, H_2_O was added to the reaction mixture. The solid precipitate, when formed, was separated by filtration and washed with H_2_O and CH_2_Cl_2_. When no precipitate was observed, the product was extracted with EtOAc. The organic extracts were washed with H_2_O and brine, dried over Na_2_SO_4_ and then evaporated to give the polyhydroxylated analogous compounds **1** to **10**. The crude products were purified as indicated in each case.

#### 2.2.1. N-(2,4-Dihydroxyphenyl)-2-(2′,5′-dihydroxyphenyl)acetamide (1)

Reddish solid, 41% yield. m.p.: 178–180 °C (Precipitated with Et_2_O). HPLC: *t_R_* = 6.06 min (10 min gradient: 15 to 95% of A in B). ^1^H-NMR (300 MHz, DMSO-*d_6_*) δ: 3.51 (s, 2H, CH_2_), 6.15 (dd, 1H, *J* = 8.6, 2.5 Hz, 4′-H), 6.29 (d, 1H, *J* = 2.6 Hz, 6′-H), 6.48 (dd, 1H, *J* = 8.5, 2.9 Hz, 5-H), 6.59 (d, 1H, *J* = 2.9 Hz, 3-H), 6.63 (d, 1H, *J* = 8.5 Hz, 6-H), 7.47 (d, 1H, *J* = 8.6 Hz, 3′-H), 8.67 (s, 1H, NH), 9.01 (s, 1H, OH), 9.03 (s, 1H, OH), 9.08 (s, 1H, OH), 9.64 (s, 1H, OH) ppm. ^13^C-NMR (75 MHz, DMSO-*d_6_*) δ: 38.2 (CH_2_), 102.9 (C3), 105.6 (C5), 114.2 (C6′), 115.8 (C4′), 117.2 (C1), 118.3 (C3′), 122.5 (C1′), 122.8 (C6), 147.7 (C), 148.7 (C), 149.8 (C), 154.5 (C), 169.5 (CO) ppm. MS (ESI^+^): *m/z* 276.4 (M+H)^+^.

#### 2.2.2. 2-(2′,4′-Dihydroxyphenyl)-N-(2,5-dihydroxyphenyl)acetamide (2)

Reddish solid, 44% yield, m.p.: 77–79 °C, (precipitated with Et_2_O). HPLC: *t_R_* = 2.50 min (10 min gradient: 15 to 95% of A in B). ^1^H-NMR (300 MHz, DMSO-*d_6_*) δ: 1.91 (s, 2H, CH_2_), 6.19 (dd, 1H, *J* = 8.2, 2.3 Hz, 4′-H), 6.27 (dd, 1H, *J* = 8.5, 2.9 Hz, 5-H), 6.33 (d, 1H, *J* = 2.3 Hz, 6′-H), 6.58 (d, 1H, *J* = 8.5 Hz, 6-H), 6.93 (d, 1H, *J* = 8.2 Hz, 3′-H), 7.49, (d, 1H, *J* = 2.9 Hz, 3-H), 8.70 (br s, 1H, OH), 8.78 (s, 1H, NH) 9.03 (br s, 1H, OH), 9.19 (br s, 1H, OH), 9.61 (br s, 1H, OH) ppm. ^13^C-NMR (75 MHz, DMSO-*d_6_*) δ: 38.3 (CH_2_), 102.9 (C3), 106.4 (C6′), 107.3 (C5), 109.8 (C1), 112.4 (C4′), 115.4 (C3′), 127.1 (C1′), 131.4 (C6), 138.8 (C), 149.8 (C), 156.0 (C), 157.5 (C), 170.0 (CO) ppm. MS (ESI^+^): *m/z* 276.4 (M+H)^+^. 

#### 2.2.3. 2-(2′,5′-Dihydroxyphenyl)-N-(4-hydroxyphenyl)acetamide (3)

White lyophilized solid, purified by column chromatography, EtOAc-Hex (gradient from 1:2 to 3:1). Yield of 79%, m.p.: 185–187 °C^d^. HPLC: *t_R_* = 2.17 min (5 min gradient: 15 to 95% of A in B). ^1^H-NMR (400 MHz, DMSO-d_6_) δ: 3.46 (s, 2H, CH_2_), 6.45 (dd, 1H, *J* = 8.3, 2.9 Hz, 4′-H), 6.58 (d, 1H, *J* = 2.8 Hz, 6′-H), 6.59 (d, 1H, *J* = 8.6 Hz, 3′-H), 6.68 (d, 2H, *J* = 8.8 Hz, 3-H, 5-H), 7.37 (d, 1H, *J* = 8.6 Hz, 2-H,6-H), 8.63 (br s, 1H, OH), 8.84 (br s, 1H, OH), 9.16 (br s, 1H, NH), 9.80 (s, 1H, OH) ppm. ^13^C-NMR: (75 MHz, DMSO-d_6_) δ: 38.0 (CH_2_), 113.9 (C4′), 115.0 (C3,C5), 115.6 (C3′), 117.1 (C6′), 120.9 (C2, C6), 123.2 (C1′), 130.9 (C1), 147.7 (C), 149.7 (C), 153.24 (C), 169.0 (CO) ppm. MS (ESI^+^): *m/z* 260.33 (M+H)^+^. 

#### 2.2.4. N-(2,5-Dihydroxyphenyl)-2-(2′,5′-dihydroxyphenyl)acetamide (4)

Reddish lyophilized solid, purified by column chromatography, EtOAc-Hex (gradient from 1:1 to 4:1). Yield of 79%, m.p.: 204–207 °C^d^. HPLC: *t_R_* = 2.24 min (5 min gradient: 15 to 95% of A in B). ^1^H-NMR (400 MHz, DMSO-d_6_) δ: 3.53 (s, 2H, CH_2_), 6.28 (dd, 1H, *J* = 8.6, 2.9 Hz, 4-H), 6.49 (dd, 1H, *J* = 8.6, 2.9 Hz, 4′-H), 6.60 (m, 2H, 3′-H,6′-H), 8.65 (d, 1H, *J* = 8.6 Hz, 3-H), 7.48 (d, 1H, *J* = 2.9 Hz, 6-H), 8.71 (s, 1H, OH), 8.73 (s, 1H, OH), 8.95 (s, 1H, NH), 0.03 (s, 1H, OH), 9.07 (br s, 1H, OH) ppm. ^13^C-NMR: (75 MHz, DMSO-d_6_) δ: 39.8 (CH_2_), 107.6 (C6), 110.0 (C3), 114.4 (C4), 115.5 (C4′), 115.7 (C3′), 117.2 (6′), 122.5 (C1′), 127.0 (C1), 138.3 (C), 147.6 (C), 149.8 (C), 149.8 (C), 169.5 (CO) ppm. MS (ESI^+^): *m/z* 276.27 (M+H)^+^. 

#### 2.2.5. N-(2,4-Dihydroxyphenyl)-2-(2′,4′-dihydroxyphenyl)acetamide (5)

White lyophilized solid, purified by column chromatography, EtOAc-Hex (gradient from 1:1 to 4:1). Yield of 94%, m.p.: 75–78 °C^d^ (MeOH). HPLC: *t_R_* = 2.24 min (5 min gradient: 15 to 95% of A in B). ^1^H-NMR (400 MHz, DMSO-d_6_) δ: 3.43 (s, 2H, CH_2_), 6.13 (dd, 1H, *J* = 8.7, 2.6, 4′-H), 6.16 (dd, 1H, *J* = 8.1, 2.4, 4-H), 6.25 (d, 1H, *J* = 2.6 Hz, 6′-H), 6.29 (d, 1H, *J* = 2.4 Hz, 6-H), 6.90 (d, 1H, *J* = 8.2 Hz, 3′-H), 7.48 (d, 1H, *J* = 8.7 Hz, 3-H), 8.84 (br s, 1H, NH), 9.12 (br s, 2H, OH), 9.51 (br s, 2H, OH) ppm. ^13^C-NMR: (75 MHz, DMSO-d_6_) δ: 37.8 (CH_2_), 102.6 (C3′), 102.9 (C3), 105. 7 (C5), 106.4 (C5′), 112.7 (C6), 118.5 (C6′), 122.2 (C1′), 131.3 (C1), 148.4 (C), 154.4 (C), 156.1 (C), 157.4 (C), 170.0 (CO) ppm. MS (ESI^+^): *m/z* 276.34 (M+H)^+^. 

#### 2.2.6. 2-(2′,5′-Dihydroxyphenyl)-N-(3,4-dihydroxyphenyl)acetamide (6)

Reddish lyophilized solid, purified by column chromatography EtOAc-Hex (gradient from 1:1 to 2:1), 56% yield, m.p.: 180 °C^d^. HPLC: *t_R_* = 4.02 min (5 min gradient: 2 to 95% of A in B) ^1^H-NMR (400 MHz, DMSO-d_6_) δ: 3.34 (s, 2H, CH_2_), 6.45 (dd, 1H, *J* = 8.5, 2.9, 5′-H), 6.57 (d, 1H, *J* = 2.9, 6′-H), 6.59 (d, 1H, *J* = 8.5 Hz, 5-H), 6.62 (d, 1H, *J* = 8.5 Hz, 4′-H), 6.79 (dd, 1H, *J* = 8.5, 2.4 Hz, 6-H), 7.14 (d, 1H, *J* = 2.4 Hz, 2-H), 8.57 (s, 1H, OH), 8.62 (s, 1H, OH), 8.85 (s, 1H, NH), 8.92 (s, 1H, OH), 9.69 (s, 1H, OH) ppm. ^13^C-NMR: (75 MHz, DMSO-d_6_) δ: 38.2 (CH_2_), 107.9 (C2), 110.4 (C6), 113.9 (C4′), 115.2 (C5), 115.6 (C3′), 117.1 (C6′), 123.2 (C1′), 131.3 (C1), 141.2 (C), 144.9 (C), 147.7 (C), 149.7 (C), 169.0 (CO) ppm. MS (ESI^+^): *m/z* 276.27 (M+H)^+^. 

#### 2.2.7. 2-(3′,4′-Dihydroxyphenyl)-N-(4-hydroxyphenyl)acetamide (7)

White lyophilized solid, purified by column chromatography EtOAc-Hex (gradient from 1:1 to 3:1), 75% yield, m.p.: 192–194 °C. HPLC: *t_R_* = 2.09 min (5 min gradient: 15 to 95% of A in B). ^1^H-NMR (400 MHz, DMSO-d_6_) δ: 3.36 (s, 2H, CH_2_), 6.54 (dd, 1H, *J* = 8.0, 2.1, 6′-H), 6.64 (d, 1H, *J* = 8.0, 3′-H), 6.66 (d, 2H, *J* = 8.9 Hz, 3-H, 5-H), 6.72 (d, 1H, *J* = 2.1 Hz, 2′-H), 7.35 (d, 2H, *J* = 8.9 Hz, 2-H, 6-H), 8.70 (s, 1H, OH), 8.81 (br s, 1H, OH), 9.14 (s, 1H, NH), 9.76 (br s, 1H, OH) ppm. ^13^C-NMR: (75 MHz, DMSO-d_6_) δ: 43.0 (CH_2_), 115.3 (C3, C5), 115.6 (C2′), 116.6 (C5′), 120.0 (C6′), 121.1 (C2, C6), 127.3 (C1′), 131.3 (C1), 144.2 (C), 145.3 (C), 153.5 (C), 169.2 (CO) ppm. MS (ESI^+^): *m/z* 260.33 (M+H)^+^.

#### 2.2.8. 2-(2′,4′-Dihydroxyphenyl)-N-(4-hydroxyphenyl)acetamide (8)

White lyophilized solid, purified by column chromatography EtOAc-Hex (gradient from 1:3 to 2:1), 80% yield, precipitated with Et_2_O (m.p.: 186–189 °C). HPLC: *t_R_* = 2.43 min (5 min gradient: 15 to 95% of A in B). ^1^H-NMR (400 MHz, DMSO-d_6_) δ: 3.45 (s, 2H, CH_2_), 6.17 (dd, 1H, *J* = 8.2, 2.4, 5′-H), 6.27 (d, 1H, *J* = 2.4, 3′-H), 6.67 (d, 2H, *J* = 8.8 Hz, 3-H, 5-H), 6.88 (d, 1H, *J* = 8.2 Hz, 6′-H), 7.36 (d, 2H, *J* = 8.8 Hz, 2-H, 6-H), 9.07 (s, 1H, NH), 9.14 (s, 1H, OH), 9.39 (br s, 1H, OH), 9.69 (br s, 1H, OH) ppm. ^13^C-NMR: (75 MHz, DMSO-d_6_) δ: 37.4 (CH_2_), 102.5 (C3), 106.1 (C5′), 113.1 (C1′), 115.0 (C3, C5), 120.9 (C2, C6), 131.0 (C-1), 131.1 (C6′), 153.2 (C), 156.1 (C), 157.1 (C), 169.6 (CO) ppm. MS (ESI^+^): *m/z* 260.33 (M+H)^+^. 

#### 2.2.9. 2-(2′,5′-Dihydroxyphenyl)-N,N-bis(4-hydroxyphenyl)acetamide (9)

White lyophilized solid, purified by column chromatography EtOAc-Hex (gradient from 1:2 to 2:1), 72% yield, precipitated with Et_2_O, m.p.: 124–126 °C. HPLC: *t_R_* = 4.57 min (5 min gradient: 2 to 95% of A in B). ^1^H-NMR (400 MHz, DMSO-d_6_) δ: 3.26 (s, 2H, CH_2_), 6.34 (dd, 1H, *J* = 8.5, 2.9, 4′-H), 6.27 (d, 1H, *J* = 2.9, 6′-H), 6.43 (d, 1H, *J* = 8.5 Hz, 3′-H), 6.62 (d, 2H, *J* = 8.1 Hz, 3-H, 5-H), 6.68 (d, 2H, *J* = 8.2 Hz, 3″-H, 5″-H), 6.98 (d, 2H, *J* = 8.2 Hz, 2″-H, 6″-H), 7.10 (d, 2H, *J* = 8.1 Hz, 2-H, 6-H), 8.51 (s, 1H, OH), 8.57 (s, 1H, OH), 9.33 (br s, 1H, OH), 9.57 (br s, 1H, OH) ppm. ^13^C-NMR: (75 MHz, DMSO-d_6_) δ: 35.7 (CH_2_), 113.6 (C3″, C5″), 115.2 (C4′), 115.3 (C3, C5), 115.9 (C3′, C6′), 117.2 (C2″, C6″), 123.4 (C2, C6), 127.8 (C-1′), 129.4 (C1″), 134.9 (C1), 147.6 (C), 149.5 (C), 155.4 (C), 156.6 (C), 170.9 (CO) ppm. MS (ESI^+^): *m/z* 352.29 (M+H)^+^. 

#### 2.2.10. N-(2′,5′-Dihydroxybenzyl)-2-(2″,5″-dihydroxyphenyl)-N-(4-hydroxyphenyl) acetamide (10) 

White lyophilized solid, purified by column chromatography EtOAc-Hex (gradient from 1:2 to 2:1), 81% yield, precipitated with Et_2_O, m.p.: 114–116 °C. HPLC: *t_R_* = 4.64 min (5 min gradient: 2 to 95% of A in B). ^1^H-NMR (400 MHz, DMSO-d_6_) δ: 3.25 (s, 2H, CH_2_), 4.66 (s, 2H, NCH_2_), 6.41–6.46 (m, 3H, 4′H, 4″H, 6″H), 6.49 (d, 1H, *J* = 2.9, 6′-H), 6.52 (d, 1H, *J* = 8.2 Hz, 3′H), 6.54 (d, 1H, *J* = 8.5 Hz, 3″H), 6.74 (d, 2H, *J* = 8.7 Hz, 3H, 5H), 7.01 (d, 2H, *J* = 8.7 Hz, (d, 2H, *J* = 8.1 Hz, 2H, 6H), 8.56 (s, 1H, OH), 8.60 (s, 1H, OH), 8.68 (s, 1H, OH), 8.73 (br s, 1H, OH), 9.63 (br s, 1H, OH) ppm. ^13^C-NMR: (75 MHz, DMSO-d_6_) δ: 35.0 (CH_2_), 48.2 (*N*-CH_2_), 113.8 (C4″), 114.6 (C6″), 115.4 (C3″), 115.8 (C3, C5), 115.9 (C4′), 117.4 (C3′, C6′), 123.1 (C1′), 123.7 (C1″), 129.1 (C2, C6), 133.7 (C1), 147.4 (C), 147.7 (C), 149.5 (C), 149.6 (C), 156.7 (C), 171.7 (CO) ppm. MS (ESI^+^): *m/z* 382.29 (M+H)^+^. 

### 2.3. Synthesis of Polyhydroxylated Ureas (11 to 18)

Treatment with BBr_3_ of the corresponding methoxylated ureas_,_ following the same procedure above described for the synthesis of amide derivatives **1** to **10**, led to the polyhydroxylated ureas **11** to **18**.

#### 2.3.1. N-(2,4-Dihydroxyphenyl)-N′-(2′,5′-dihydroxyphenyl)urea (11)

Whitish lyophilized solid, 95% yield, precipitated with Et_2_O, m.p.: >155 °C^d^. HPLC: *t_R_* = 5.97 min (15 min gradient: 2 to 40% of A in B). ^1^H-NMR (500 MHz, DMSO-*d_6_*) δ: 6.16 (dd, 1H, *J* = 8.6, 3.0 Hz, 4′-H), 6.18 (dd, 1H, *J* = 8.5, 2.9 Hz, 5-H), 6.31 (d, 1H, *J* = 2.7 Hz, 3-H), 6.58 (d, 1H, *J* = 8.5 Hz, 3′-H), 7.43 (d, 1H, *J* = 8.7 Hz, 6-H), 7.46 (d, 1H, *J* = 2.9 Hz, 6′-H), 8.45 (s, 1H, NH), 8.47 (s, 1H, NH), 8.59 (br s, 1H, OH), 8.93 (br s, 1H, OH), 9.00 (s, 1H, OH), 9.58 (s, 1H, OH) ppm. ^13^C-NMR (125 MHz, DMSO-*d_6_*) δ: 102.7 (C-3), 105.6 (C6′), 106.7 (C4′), 107.6 (C5), 115.0 (C3′), 119.0 (C1), 122.3 (C6), 128.6 (C1′), 138.4 (C), 148.5 (C), 149.9 (CO), 153.4 (C), 153.6 (C) ppm. MS (ESI^+^): *m/z* 277.3 (M+H)^+^. 

#### 2.3.2. N-(2,5-Dihydroxyphenyl)-N′-(4′-hydroxyphenyl)urea (12) 

White prisms, 56% yield, m.p.: 180–182 °C (MeOH). HPLC: *t_R_* = 2.49 min (10 min gradient: 15 to 95% of A in B). ^1^H-NMR (400 MHz, DMSO-*d*_6_) δ: 6.15 (dd, 1H, *J* = 8.4, 2.8 Hz, 4-H), 6.59 (d, 1H, *J* = 8.5 Hz, 3-H), 6.67 (d, 2H, *J* = 8.8 Hz, 3′-H, 5′-H), 7.20 (d, 2H, *J* = 8.8 Hz, 2′-H, 6′-H), 7.58 (d, 1H, *J* = 2.8 Hz, 6-H), 7.94 (d, 1H, NH), 8.60 (s, 1H, NH), 8.97 (s, H, OH), 9.03 (s, 1H, OH), 9.1 (s, 1H, OH) ppm. ^13^C-NMR: (75 MHz, DMSO-*d*_6_) δ: 106.2 (C6), 107.3 (C4), 114.8 (C3), 115.3 (C3′, C5′), 120.1 (C2′, C6′), 128.7 (C1), 131.5 (C1′), 137.9 (C), 150.0 (C), 152.4 (CO), 152.7 (C), ppm. MS (ESI^+^): *m/z* 261.28 (M+H)^+^.

#### 2.3.3. N-(2,5-Dihydroxyphenyl)- N′-(3′,4′-dihydroxyphenyl)urea (13) 

White prisms, 68% yield, m.p.: 141–143 °C (Cl_2_CH_2_/MeOH). HPLC: *t_R_* = 1.73 min (10 min gradient: 15 to 95% of A in B). ^1^H-NMR (400 MHz, DMSO-d_6_) δ: 6.1-6.2 (m, 2H, 4,6′-H), 6.31 (d, 1H, *J* = 2.6, 2′-H), 6.58 (d, 1H, *J* = 8.5 Hz, 3-H), 7.43 (d, 1H, *J* = 8.7 Hz, 5′-H), 7.46 (d, 1H, *J* = 2.9 Hz, 6-H), 8.44 (s, 1H, NH), 8.47 (s, 1H, OH), 8.59 (s, 1H, OH), 8.94 (s, 1H, OH), 9.01 (s, 1H, OH), 9.59 (s, 1H, NH) ppm. ^13^C-NMR: (75 MHz, DMSO-d_6_) δ: 102.8 (C6), 105.6 (C2′), 106.8 (C4), 107. 7 (C5′), 115.1 (C3), 119.0 (C6′), 122.4 (C1), 128.6 (C1′), 138.4 (C), 148.6 (C), 149.9 (C), 153,4 (C), 153.6 (CO) ppm. MS (ESI^+^): *m/z* 277.29 (M+H)^+^.

#### 2.3.4. N-(3,4-Dihydroxyphenyl)-N′-(4′-hydroxyphenyl)urea (14) 

White prisms, 60% yield, m.p.: 184–187 °C (MeOH/Cl_2_CH_2_). HPLC: *t_R_* = 2.12 min (10 min gradient: 15 to 95% of A in B). ^1^H-NMR (400 MHz, DMSO-d*_6_*) δ: 6.14 (dd, 1H, *J* = 8.7, 2.6 Hz, 6-H), 6.32 (d, 1H, *J* = 2.6 Hz, 2-H), 6.66 (d, 1H, *J* = 8.8 Hz, 3′-H, 5′-H), 7.18 (d, 1H, *J* = 8.8 Hz, 2′-H, 6′-H), 7.59 (d, 1H, *J* = 8.7 Hz, 5-H), 7.68 (d, 1H, OH), 8.72 (s, 1H, OH), 8.87 (s, 1H, NH), 8.99 (s, 1H, NH), 9.69 (s, 1H, OH) ppm. ^13^C-NMR: (75 MHz, DMSO-d*_6_*) δ: 102.5 (C2), 105.5 (C5), 115.2 (C3′,5′), 119.61 (C6), 120.0 (C2′, C6′), 120.6 (C1), 131.7 (C1′), 147.2 (C), 152.2 (C), 152.7 (CO), 153.2 (C) ppm. MS (ESI^+^): *m/z* 261.31 (M+H)^+^.

#### 2.3.5. N,N′-bis(4-Hydroxyphenyl)urea (15) 

White prisms, 84% yield, m.p.: 245 °C^d^ (MeOH; m.p. Lit [17] = 240 °C^d^). HPLC: *t_R_* = 2.59 min (10 min gradient: 15 to 95% of A in B). ^1^H-NMR (400 MHz, DMSO-d_6_) δ: 6.66 (d, 4H, *J* = 8.8, 3-H, 5-H), 7.18 (d, 4H, *J* = 8.8 Hz, 2-H, 6-H), 8.16 (s, 2H, NH), 8.99 (s, 2H, OH), ppm. ^13^C-NMR: (75 MHz, DMSO-d_6_) δ: 115.2 (C3, C5), 120.3 (C2, C6), 131.5 (C1), 152.3 (C4), 153.1 (CO), ppm. MS (ESI^+^): *m/z* 245.30 (M+H)^+^. 

#### 2.3.6. N,N′-bis(2,5-Dihydroxyphenyl)urea (16) 

Lyophilized solid, purified by column chromatography EtOAc-Hex (gradient from 1:3 to 4:1), 20% yield, m.p.: 109 °C^d^. HPLC: *t_R_* = 1.76 min (10 min gradient: 15 to 95% of A in B). ^1^H-NMR (500 MHz, DMSO-d_6_) δ: 6.19 (dd, 2H, *J* = 8.5, 2.9 Hz, 4-H), 6.59 (d, 2H, *J* = 8.5 Hz, 3-H), 7.47 (d, 2H, *J* = 2.9 Hz, 6-H), 8.6 (s, 1H, NH), 8.74 (s, 2H, OH), 8.99 (s, 2H, OH) ppm. ^13^C-NMR: (125 MHz, DMSO-d_6_) δ: 107.2 (C6), 108.0 (C4), 115.1 (C3), 128.4 (C1), 138.7 (C2), 149.9 (C5), 153.0 (CO) ppm. MS (ESI^+^): *m/z* 277.39 (M+H)^+^. 

#### 2.3.7. N,N-bis(4-Hydroxyphenyl)-N′-(4′-hydroxyphenyl)urea (17) 

Lyophilized solid, purified by column chromatography EtOAc-Hex (gradient from 1:2 to 3:1), 68% yield, m.p.: 186–187 °C^d^. HPLC: *t_R_* = 7.15 min (10 min gradient: 2 to 95% of A in B). ^1^H-NMR (400 MHz, DMSO-d_6_) δ: 6.60 (d, 2H, *J* = 8.9, 3′-H, 5′-H), 6.72 (d, 4H, *J* = 8.8, 3-H, 5-H), 7.05 (d, 4H, *J* = 8.7 Hz, 2-H, 6-H), 7.12 (d, 2H, *J* = 8.9 Hz, 2′-H, 6′-H), 7.41(s, 1H, NH), 9.02 (s, 1H, OH), 9.43 (s, 2H, OH) ppm. ^13^C-NMR: (75 MHz, CDCl_3_) δ: 114.7 (C3′, C5′), 115.7 (C3, C5), 122.3 (C2′, C6′), 128.7 (C2, C6), 131.2 (C), 134.9 (C), 152.8 (C), 154.6 (CO), 155.4 (C) ppm. MS (ESI^+^): *m/z* 337.32 (M+H)^+^.

#### 2.3.8. N-(2′,5′-Dihydroxybenzyl)- N′-(2″,5″-dihydroxiphenyl)-N-(4-hydroxyphenyl)urea (18) 

White lyophilized solid, purified by column chromatography EtOAc-Hex (gradient from 1:3 to 4:1), 10% yield, m.p.: 114-115 °C (MeOH). HPLC: *t_R_* = 5.40 min (10 min gradient: 15 to 95% of A in B). ^1^H-NMR (400 MHz, DMSO-d_6_) δ: 4.67 (s, 2H, *N*-CH_2_), 6.14 (dd, 1H, *J* = 8.5, 2.8, 4″-H), 6.43–6.48 (m, 2H, 4′-H, 3′-H), 6.56 (d, 1H, *J* = 8.6 Hz, 3″-H), 6.59 (d, 1H, *J* = 2.8 Hz, 6′-H), 6.80 (d, 1H, *J* = 8.7 Hz, 3-H, 5-H), 7.05 (s, 1H, NH), 7.10 (d, 1H, *J* = 8.7 Hz, 2-H, 6-H), 7.57 (d, 1H, *J* = 2.8 Hz, 6″-H), 8.62 (br s, 1H, OH), 8.65 (br s, 1H, OH), 8.78 (br s, 1H, OH), 8.88 (br s, 1H, OH), 9.72 (br s, 1H, OH) ppm. ^13^C-NMR: (100 MHz, DMSO-d_6_) δ: 47.9 (*N*-CH_2_), 105.6 (C6″), 107.6 (C6′), 114.4 (C4″), 114.5 (C4′), 115.3 (C3″), 115. 9 (C3′), 116.5 (C3, C5), 124.7 (C), 128.2 (C), 129.5 (C2, C6), 132.0 (C), 137.5 (C), 147.2 (C), 149.7 (C), 150.0 (C), 154.4 (CO), 156.9 (C) ppm. MS (ESI^+^): *m/z* 383.25 (M+H)^+^.

### 2.4. Antioxidant Activity 

#### 2.4.1. Oxygen Radical Absorbance Capacity (ORAC) Experiment

The ORAC assay was performed following Ou et al. [18], as modified by Garcés-Rimón et al. [19]. All samples and reagents were dissolved in phosphate buffer (75 mM; pH 7.4). The reaction was performed in a final volume of 200 μL: 20 μL test samples or 20 μL 6-hydroxy-2,5,7,8-tetramethylchroman-2-carboxylic acid (Trolox) solutions (0.2–2 nM), 120 μL fluorescein solution (1.17 mM) and 60 μL 2,2′-azo-bis-(2-methylpropionamidine) dihydrochloride (AAPH) 1.3% solution (all from Sigma Aldrich, Alcobendas, Spain) were added to the wells of a black 96-well plate. The fluorescence was recorded at 40 °C every 55 s for 95 min using a fluorimeter (SpectraMax M2; Molecular Devices, California, USA), with excitation and emission wavelengths of 480 and 520 nm, respectively. All samples were tested in triplicate. ORAC values were expressed as μmol of Trolox equivalents (TE)/μmol of pure compound. 

#### 2.4.2. ABTS Experiment

The 2,2′-azino-bis (3-ethylbenzothiazoline-6-sulfonic acid) diammonium salt (ABTS) assay was performed according to Re et al. [20] and modified by Oki et al. [21] for its use in microplates. Samples were diluted in methanol. An ABTS^•+^ stock solution was prepared by adding 44 μL of potassium persulfate (140 mmol/L) to a 2.5 mL ABTS^•+^ aqueous solution (7 mmol/L). The working solution of the radical ABTS^+^ was prepared by diluting the stock solution 1:75 (*v/v*) in a sodium phosphate buffer (5 mmol/L, pH 7.4) to obtain an absorbance value of 0.7±0.02 at 734 nm. Samples (30 μL) were added to 270 μL of the working solution of ABTS^•+^ in a microplate. Absorbance was measured at 734 nm and 30 °C for 20 min, every 5 min in a Synergy HT plate spectrophotometer (Biotek Instruments, Winoosky, VT, USA). A calibration curve was made with Trolox (20–250 μM). All samples were analyzed in triplicate. Results were expressed in μmol TE/μmol of pure compound, as for the ORAC assays. 

#### 2.4.3. DPPH Experiment

For the 2,2′-diphenylpicrylhydrazyl (DPPH) assay, antioxidant compounds were dissolved in ethanol with a stock concentration of 4 mM and stirred for 24 h. DPPH (Alfa Aesar) was also dissolved in ethanol at 0.127 mM. Different compound solutions were prepared by serial dilutions of each stock solution (4, 2, 1, 0.5, 0.25, 0.125, 0.063, 0.031, 0.015, and 0.007 M). Resveratrol was prepared using the same protocol and used as antioxidant control. Amounts of 80 µL of each solution and 80 µL of DPPH were added in a 96-well plate. The absorbance was measured at different times (10, 20 and 30 min and 1 and 2 h) by a Multi-Detection Microplate Reader Synergy HT (λ_MAX_ = 515 nm). Radical scavenging activity (RSA, %) was calculated using Equation (1)

(1) $$\mathrm{RSA}(\%)=\frac{A_{\mathrm{DPPH}}-A_{\mathrm{EXTRACT}}}{A_{\mathrm{DPPH}}}\cdot 100$$

where A_EXTRACT_ and A_DPPH_ correspond to the absorbance of DPPH with and without the extracts, respectively. Eight replicates were used for each compound and results were expressed as mean value ± standard deviation. The RSA half-maximal inhibitory concentration (IC_50_) values were calculated from the relationship curve of RSA versus concentrations by a non-linear fit using the software GraphPad Prism 7.

### 2.5. In Vitro Cellular Assays 

#### Cell Viability

Cytotoxicity of selected antioxidant compounds was assessed using human dermal fibroblasts (HDF, Innoprot, P10856) and an AlamarBlue assay. Human fibroblasts were grown and maintained using Dulbecco’s Modified Eagle Medium high glucose (DMEM, Sigma Aldrich, D6171), supplemented with 10% Fetal Bovine Serum (FBS), 2% L-glutamine and 1% penicillin/streptomycin. Cells were incubated at 37 °C, 95% relative humidity and 5% CO_2_. Additionally, DMEM-high glucose, HEPES, no phenol red (Gibco, 2106329) was used to prepare the AlamarBlue solution. Stock solutions of the antioxidant compounds **3**, **8**, **15** and **17** were prepared in dimethyl sulfoxide (DMSO). Compound **7** was not soluble enough in DMSO to obtain a cell viability IC_50_ value. Serial dilutions of the antioxidant compounds were prepared using the previously mentioned stock solutions and DMEM. Final DMSO concentration was maintained lower than 1% *v/v* in the cell culture experiments. HDF were seeded at 9 × 10^4^ cells/well in a 96-well plate and incubated for 24 h. The medium was replaced by the corresponding antioxidant solutions and incubated for an additional 24 h. Then, compounds were removed, cells were washed with Dulbecco’s Phosphate Buffered Saline (PBS, Sigma Aldrich) and treated with a 10% *v/v* AlamarBlue (Invitrogen) solution prepared in DMEM without phenol red. After 3 h of incubation, absorbance was monitored at 570 nm by a Multi-Detection Microplate Reader Synergy HT. Cells treated with DMEM without any antioxidant compound were used as the 100% viability control. Eight replicates were used for each antioxidant solution. IC_50_ values were obtained by a non-linear fit using the software GraphPad Prism 7.

### 2.6. Reduced Nicotinamide Adenine Dinucleotide Phosphate (NADPH) Oxidase Activity Assay in Vascular Systems

The human microvascular endothelial cells line (HMEC-1) (ATCC^®^, Middlesex, UK; CRL-3243™) was used. Cells were cultured according to the manufacturer instructions with MCDB131 medium (Corning, NY, USA, Cat. No. 702564) supplemented with 10 ng/mL epidermal growth factor (Sigma-Aldrich, Alcobendas, Spain), 1 μg/mL hydrocortisone (Sigma-Aldrich), 10 mmol/L glutamine (Sigma-Aldrich), 10% fetal bovine serum (FBS, Sigma-Aldrich), 100 U/mL of penicillin and 100 µg/mL of streptomycin. At 80% confluence, cells were serum-deprived for 24 h before stimulation. HMEC were treated with AngII (1 nmol/L for 6 h; Sigma-Aldrich) in the absence or in the presence of the different compounds that were added 30 min before stimulation.

Aortic segments from three-month-old C57BL6/J mice were incubated with IL-1β (10 ng/mL, 6 h, Sigma Aldrich) in the presence and in the absence of the different compounds that were added 30 min before stimulation. 

The O_2_ production generated by NADPH oxidase was determined by a chemiluminescence assay. Briefly, endothelial cells or aortic segments were homogenized in phosphate buffer (50 mmol/L KH_2_PO_4_, 1 mmol/L EGTA, 150 mmol/L sucrose, pH 7.4). The reaction was started by the addition of a lucigenin (5 μmol/L) and NADPH (100 μmol/L; Sigma-Aldrich) mixture to the protein sample in a final volume of 250 μL. Chemiluminescence was determined every 2.4 s for 3 min in a microtiter plate luminometer (Enspire Perkin Elmer). Basal activity in the absence of NADPH was subtracted from each reading and normalized to protein concentration. Data were normalized vs. control situation. 

All data are expressed as mean values ± standard mean error and n represents the number of animals or different cell cultures. Statistical analysis was performed using GraphPad Prism Software (v7.04). Data distribution (by Shapiro–Wilk normality test) was used to choose the appropriate statistics test. Results were analyzed by the Mann–Whitney non-parametric or Student’s t-tests when appropriate (two-tailed) or one-way Anova or Kruskal–Wallis test when appropriate followed by Bonferroni’s post hoc test or uncorrected Dunn′s test, respectively.

### 2.7. In Vivo Antioxidant Capacity in Stressed Yeasts 

#### 2.7.1. Yeast Strain and Induction of Oxidative Stress

*Saccharomyces cerevisiae* haploid strain BY4741 was used as described previously [16,22]. Briefly, 20 μL of glycerol stock culture was revived in 5ml of YPD medium (1% yeast extract, 2% bacteriological peptone and 2% dextrose) at 28 °C, with shaking (210 rpm). Fifty microliters of 20-h-old culture was used to inoculate fresh 5ml YPD (control medium) or 5 mL of oxidative stress medium (YPOxD: YPOxD; 1% yeast extract, 2% bacteriological peptone, 1% dextrose and 3% H_2_O_2_). Stress (and control) was conducted during 16h at 28 °C, with shaking (220 rpm). 

#### 2.7.2. Measurement of Antioxidant Capacity

In vivo antioxidant capacity was determined as recovery of growth rate in stressed yeasts (yeast grown in YPOxD for 16h) challenged by compounds **8**, **15** and **17** (dissolved in methanol at 2 mg/mL), and compared to Resveratrol and vitamin C (dissolved in methanol at 2 mg/mL) as positive controls at four different doses (2, 10, 20 and 40 μg of each pure compound) or compared to stressed cells directly, as previously described [16,23]. Growth rate was determined from growth kinetics using the BioScreen C plate-reader system (OY Growth Curves Ab Ldt., Helsinki, Finland). Stressed yeast cultures (16 h old) were used to inoculate 200 µl of fresh media to an initial OD_595_ of 0.05–0.07, being distributed in 100-well honeycomb plates (Oy Growth Curves) in triplicate per each condition (each different dose of each compound). Plates were then incubated at 28 °C for 72h, with shaking (High force), taking OD measurement each 15 min in the Bioscreen C. Maximum growth rate (μ_max_) and biomass production (carrying capacity) were determined in R using ‘Growthcurver’ package version 0.3 [24]. Growth curves were constructed in EXCEL, represented as mean ± SEM (standard error of mean). One-way ANOVA followed by Tukey’s multiple comparisons test was performed with PRISMA v 6.0 software to compare among maximum growth rate or carrying capacity between tested compounds, Resveratrol, Vitamin C or control yeasts (grown in YPD or in YPOxD). In addition, to measure survival after oxidative stress and antioxidant effectiveness of each compound, yeast cells from each challenge were serially diluted in 96-well plates, and each dilution was spotted (with a 8 × 6 stainless steel replica platter from SIGMA) in triplicate to determine viable colonies after 24–48 h at 28 °C. Survivors (mean ± SEM) were compared between treatments with one-way ANOVA, Fisher’s LSD test, with alpha at 0.05 for significance. 

## 3. Results and Discussion

### 3.1. Preparation of Designed Polyphenols 

**Scheme 1:**
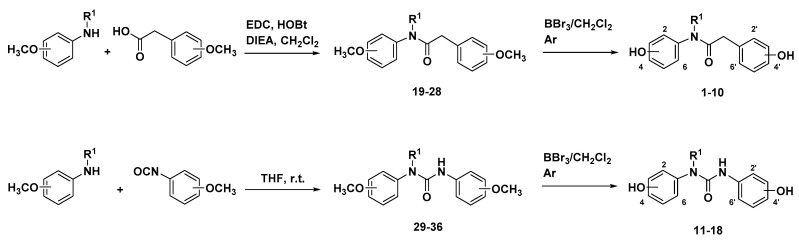
Synthetic route to polyhydroxy amides **1** to **10**, and polyhydroxy ureas **11** to **18**.

As indicated, two series of compounds were designed, both containing two polyphenol rings separated by a three-atoms linker, either containing an amide or a urea moiety. Combinations of 4-, 2,4-, 3,4-(catechol) and 2,5-OH were selected based on our previous results on the triazole series [16], and by their incidence in many polyphenols from natural sources. The synthesis of the amide and urea polyhydroxyphenyl derivatives **1** to **18** was performed following conventional methods, as depicted in Scheme 1. 

The methoxyphenyl amide derivatives **19** to **28** were prepared by reaction of the conveniently substituted aniline and the corresponding phenylacetic acid, in CH_2_Cl_2_, using 1-ethyl-3-(3-dimehylaminopropyl) carbodiimide (EDC^.^HCl) and 1-hydroxybenzotriazole (HOBt) as coupling agents, in the presence of *N,N*-diisopropylethylamine (DIEA) as a base. Further treatment of methoxy-substituted intermediates **19** to **28** with BBr_3_ led to the desired hydroxylated amides, compounds **1** to **10** (Scheme 1). On the other hand, the preparation of the ureido compounds **29**–**36** was carried out from the corresponding methoxyanilines and the methoxy-substituted isocyanates, by reaction at room temperature in tetrahydrofuran (THF) (Scheme 1). The final hydroxyl derivatives (**11** to **18**) were prepared from **29** to **36** also by demethylation reaction with BBr_3_. The synthesis proceeded very smoothly and most compounds were obtained in moderate-to-good yields with elevated grade of purity (Table 1 and Table 2). The acylation reaction was especially difficult in the case of the secondary bis-4-methoxyphenylamine to obtain methoxy amide **27**, and during the urea formation of intermediate **36**. Low yields in some demethylation reactions seem to be related to low stability of the final polyhydroxylated compounds (as explained later).

### 3.2. Antioxidant Characterization of the New Polyphenols Collection

The antioxidant capacity of the small collection of new polyphenols (Table 1) was firstly evaluated in an Oxygen Radical Absorbance Capacity (ORAC) assay (Table 3). All compounds exhibited strong radical scavenging activities; 14 out of the 18 compounds tested, showed values even higher than those found for resveratrol (own data), a well-known natural antioxidant used as control. Compared to resveratrol (TE 8 µmol trolox/µmol, TE: Trolox Equivalents), six of the compounds show 2–3-fold higher antioxidant capacity. Thus, some of the results found were quite outstanding, as in the case of compounds **9** and **10** that showed values above 25 TE per sample, and **3**, **7**, **8** and **17** with >19 TE. Compounds **1**, **4**, **12**, **16**, and **18** still display higher antioxidant activity than resveratrol, while derivatives **2**, **6**, **13** and **15** are comparable to the control, and only polyphenols **5** and **11** have lower antioxidant ability compared to resveratrol (Table 3). 

Going into more detail through the data from the ORAC assay, and taking into consideration the chemical structure, we firstly focused on the compounds having only two phenyl rings in their structure. In general, it can be said that amide derivatives showed higher TE values than ureas. Comparing compounds with the same pattern of hydroxyl substituents, TE values showed by amides **1**, **3**, **7** and **8**, are higher than those of their corresponding urea derivatives **11**, **12**, **13** and **14**. In the case of **8** and **14**, the value was the double for the former. Besides, for the three best amides, **3**, **7** and **8**, it is remarkable that all of them have a *p*-monohydroxyphenyl ring at the N-amide atom, while the benzyl ring has two hydroxyl groups in 2,5-, 2,4- or 3,4- positions, respectively (Table 3). 

On the other hand, both in the case of amide and urea derivatives, the antioxidant capacity was increased by incorporation of a third phenolic ring at N position, like in **9**, **10**, **17** and **18** compounds. A 4-hydroxyphenyl substitution (**9** and **17**) led to slightly better TE values than a 2,5-dihydroxybenzyl moiety (compounds **10** and **18**). 

ORAC values for most of the prepared compounds were also higher than those found for other natural antioxidants, like quercetin, hydrotyrosol, D-catechin and (-)-epicatechin (Table S1).

Considering the high tendency to oxidation of polyphenols, and before further characterization of this series of compounds, we decided to check the stability in aqueous media of all polyphenol derivatives showing higher antioxidant capacity than resveratrol (Table 3). To facilitate the solubility, compounds were dissolved in a mixture of H_2_O/acetonitrile (80:20%) and maintained at room temperature. The solutions were checked by HPLC-MS at regular intervals of time for 60 days. Only amides **3**, **7**, **8** and ureas **15** and **17** proved to be sufficiently stable after two months. The rest of the compounds suffered progressive degradation, with complete disappearance from the solution after 10 or 20 days. The most unstable polyphenol derivatives were **4** and **10** (Figure S1), having both two p-dihydroxy substitutions in the phenyl rings, which points to the rapid and easy oxidation to the p-hydroquinone. From the four compounds with three phenyl rings, only one was stable in aqueous solution (**17**). 

After checking the chemical stability, only the five more stable compounds (**3**, **7**, **8**, **15** and **17**) were selected to complete their characterization as antioxidants. For this purpose, we used ABTS and DPPH assays, two spectrophotometric methods normally applied to show the radical scavenging ability of antioxidant compounds (Table 4). First, the compounds were evaluated in the ABTS (2,2′-azinobis-(3-ethylbenzothiazoline-6-sulfonic acid) assay [20]. In the presence of oxidant agents, ABTS form a stable radical cation (ABTS^•+^) chromophore, with an absorbance band at 734 nm. The presence of this radical cation is reduced by hydrogen-donating compounds, like polyphenols, producing a discoloration of the solution proportional to the amount of antioxidant. In this assay, four out of five compounds showed TE values higher than resveratrol (Table 4), with amide derivative **7** as the best radical scavenger (nearly fourfold higher than control). However, urea **15**, which has already the lowest ORAC value among the selected compounds, was worse than resveratrol in this assay. The radical scavenging ability of compounds **7** and **8** is higher and comparable, respectively, to those of quercetin and (-)-epicatechin natural products in this ABTS assay (Table S1).

**Figure 1:**
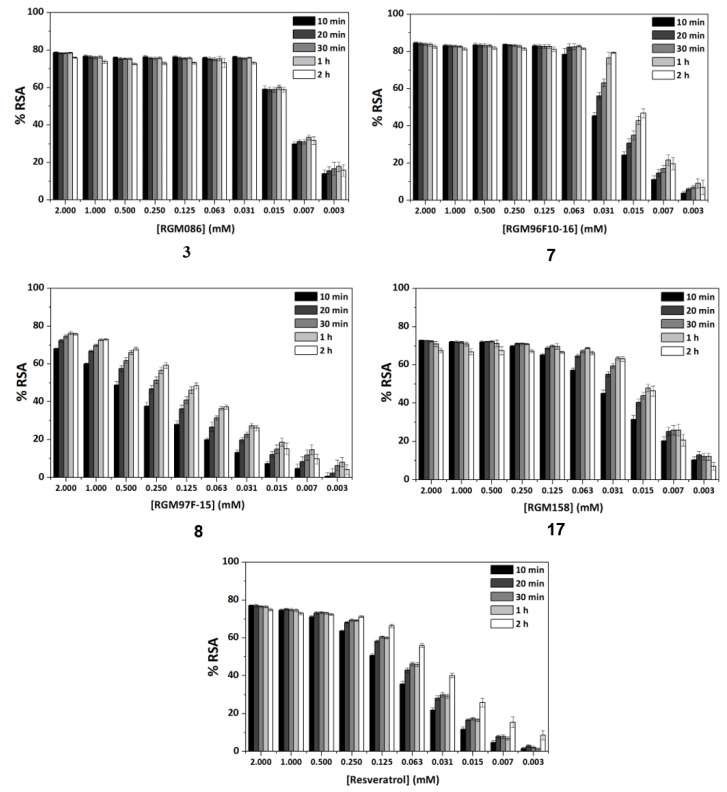
Study of the radical scavenging activity (RSA) capacity showed by compounds **3**, **7**, **8** and **17** and control (resveratrol) low in the DPPH assay. Variations with sample concentration and time.

Another frequently used method for antioxidant determination is the DPPH assay, based on the capacity of the 2,2-diphenyl-1-picryl-hydrazyl, DPPH free radical to be reduced in the presence of an antioxidant [25]. According to the previous results, only four compounds, three amides, **3**, **7** and **8**, and the urea derivative **17** were evaluated in this assay, always using resveratrol as control. The antioxidant capacity was measured at different concentrations in a progressive descending order, going from 2 to 0.003 mM, and additionally, at an interval of time for each concentration (five measures from 10 min to 2 h). For all of them, a concentration dependent behavior was observed (Figure 1). Comparing the maximum antioxidant capacities observed at the highest concentration (2 mM), at the beginning of the experiment (10 min time) the four compounds showed RSA percentage values comparable to the control, resveratrol (77%). Compounds **3** and **17**, reached about the same values as control, 78% and 72%, respectively, **8** showed a slightly lower percentage of 67%, and amide **7** proved to have a slightly higher capacity than resveratrol, 82% (Figure 1). As for the potency, the IC_50_ values shown in Table 4 indicate that the best compound in this assay is amide **3**, followed by urea **17** and amide **7**. Compared to resveratrol, the RSA capacity after 10 min was 8-, 2.8- and 3.7-fold higher for **3**, **7** and **17**, respectively. 

Looking at how the RSA capacity of compounds is modified with time (Figure 1), resveratrol (right graph) maintained the RSA capacity at concentrations above 0.5 mM. However, at lower concentrations the RSA percentages clearly diminished as a function of concentration, but increased with time (RSA 2h > RSA 10 min). In our case, compound **8**, shows this time-dependent behavior already from high concentrations (Figure S2). However, compounds **3**, **7** and **17** maintained the RSA ability nearly at the same percentage levels, independently on time, until quite low concentrations, 0.031, 0.063 and 0.25 mM, respectively, which is a remarkable difference with resveratrol (Figure 1). This behavior of **3**, **7** and **17**, will allow the use of low concentrations of sample for achieving a high effect. On the other hand, like for resveratrol, and with the exception of compound **3**, at the low concentration of 0.031 mM for **7** and **17**, the RSA ability increased with time (Figure 1). 

Compound **3**, at a concentration of 0.031 mM, still shows a remarkably high RSA (78%), which is maintained with time, from 10 min to 2 h. This time-independent behavior is observed even at lower concentrations (0.015–0.003 M), which can be an advantage for those applications that require an immediate antioxidant effect.

### 3.3. Biological Evaluation of Synthetic Polyphenols

#### 3.3.1. Cell Viability

To evaluate the potential cytotoxicity of the selected compounds, an AlamarBlue assay was performed and the cell viability IC_50_ values were extrapolated using a non-linear fit. Compound **15** crystallized when in contact with DMEM and did not allow a correct absorbance measurement in the microplate reader. For compounds **3**, **8**, and **17**, IC_50_ values were 0.246, 1.411 and 1.789 mM, respectively (Table S3, Figure S2). Compounds **17** and **8** showed a similar IC_50_, both of them over 1.4 mM, while **3** was slightly more cytotoxic, having an IC_50_ value about 5–7-fold lower (0.24 mM). Nonetheless, considering the high antioxidant capacity shown by compounds **3**, **8** and **17** with RSA IC_50_ values in the 10 min DPPH assay of 8.30, 150.90 and 17.92 µM, respectively, the concentrations needed to produce 50% of cytotoxicity are at least one order of magnitude higher than those required to reduce 50% RSA for amides **3** and **8**, and two orders greater in the case of urea **17**. Compound **7** did not show any significant cytotoxic effects at the range of concentrations where the compound was soluble in DMSO (up to 6 mM). In general, a non-cytotoxic behavior can be assured in a range of concentrations where the antioxidant compounds exhibit RSA potential. Therefore, the compounds were progressed to further, parallel biological characterization in different systems.

#### 3.3.2. Antioxidant Activity in Vascular Systems 

First, we tested the ability of two of the best antioxidant compounds, **3** and **7**, on NADPH oxidase activity in human endothelial cells stimulated with Angiotensin II, one of the most important mediators of oxidative stress and vascular damage [26]. NADPH oxidase enzymes are the major producers of ROS at the vascular level and one of the protective effect of polyphenols versus the oxidative stress is mediated by the inhibition of the expression and activity of the NADPH oxidase complex [5]. Compared to the control antioxidant resveratrol, compound **3** and **7** showed high ORAC (>19 vs. 8 TE) and ABTS (3.2 and 8.1 vs. 2.1 TE, respectively) figures, and better and comparable DPPH values, respectively (Table 4).

**Figure 2:**
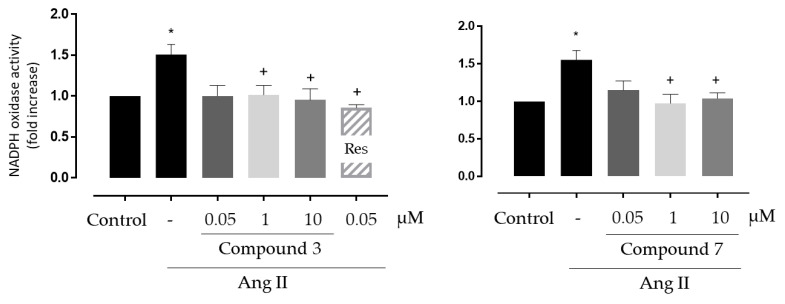
Effect of compounds **3** and **7** on Nicotinamide Adenine Dinucleotide Phosphate (NADPH) oxidase activity induced by Angiotensin II (Ang II) in cultured human microvascular endothelial cells. * *p* < 0.05 vs. control, + *p* < 0.05 vs. Ang II. Res: resveratrol (dashed bars) was used as positive control. N = 8 for compound **3** and n = 7 for compound **7**.

As shown in Figure 2, compounds **3** and **7** were able to protect the cells against the oxidative stress promoted by Angiotensin II, decreasing NADPH oxidase activity levels in the same order as resveratrol (used as control). No remarkable differences were observed between the activities of both compounds.

**Figure 3:**
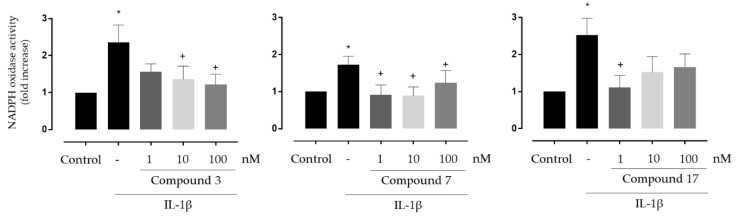
Effect of compounds **3**, **7** and **17** on NADPH oxidase activity induced by IL-1β in segments of aorta from C57BL6/J mice. * *p* < 0.05 vs. control, + *p* < 0.05 vs. IL-1β. n = 9 for compound **3**, n = 8 for compound **7** and n = 7 for compound **17**.

To further confirm the beneficial effects of these compounds in oxidative stress models at the vascular level, their antioxidant capacity was evaluated in an ex vivo whole organ system. Thus, we tested the effects of compounds **3** and, **7** on NADPH oxidase activity in mice aorta stimulated ex vivo with IL-1β, another inducer of vascular NADPH oxidase expression and activity in vascular cells [27]. Additionally, considering the good results obtained for **17** in the ORAC, ABTS and DPPH antioxidant assays, and its low toxicity, we decided to extend the study to this compound in the aorta experiment. As shown in Figure 3, IL-1β increased NADPH oxidase activity under ex vivo conditions, whereas all the three tested compounds showed significant inhibition of the IL-1β effects. 

Altogether, our results demonstrate in vitro and ex vivo antioxidant capacity of the selected compounds and suggest that they are versatile antioxidants in response to a variety of stimuli (i.e., Ang II and IL-1β) important in cardiovascular disease at the clinical level. Eventually, these compounds might be used in the treatment of vascular alterations such as endothelial dysfunction, vascular smooth muscle cells proliferation and migration, extracellular matrix remodeling, or inflammation which depend on oxidative stress milieu and that are characteristic of a number of vascular diseases including hypertension, obesity, atherosclerosis or abdominal aortic aneurysms.

#### 3.3.3. In Vivo Antioxidant Activity of Selected Compounds in Yeasts

Bioavailability, in vivo reactivity and stability, and tissue differential storage are compound properties not associated with in vitro antioxidant assays, increasing the difficulties of correlating between in vitro and in vivo transfer of activities. Therefore, we required in vivo evidence supporting the in vitro-based antioxidant activity of our compounds. In this respect, *Saccharomyces cerevisiae* is a model organism used to investigate oxidative stress and the effect of nutraceuticals in resistance to oxidative stress [16,28,29,30], among many other biological processes linked to human health. Actually, the yeast *S. cerevisiae* had become a model for the study of effects of nutraceuticals and its transfer to humans due to the new EU regulation (EC 1924/2006), because it is easy to handle, millions of cells can be studied in a single-small culture, and its genetics, biochemistry and physiology are transferrable. Indeed, yeast and humans share one in four genes as orthologues, both in sequence and in function (as reviewed in [31,32,33]). Moreover, several studies have characterized the interaction of L-ascorbic acid (vitamin C) or resveratrol, two compounds with a variety of antioxidant properties, with endogenous cellular antioxidative defense systems in *S. cerevisiae* yeast strains, which will aid in the comparison of results [32,34].

In this study, we have performed experiments with *S. cerevisiae* strain BY4741 to assess the putative in vivo antioxidant effects of compounds **8**, **15** and **17**, by assessing the growth recovery ability of yeast cells subjected to oxidative stress for at least 16h, as described previously [16]. These three compounds were selected by their differential behavior in the three in vitro antioxidant experiments. Compounds **8** and **17** show higher ORAC activity than resveratrol (used as control), while **15** is comparable to the natural product (Table 4). In the ABTS assay, urea **15** shows a lowest value than resveratrol, while the other two polyphenolic derivatives have higher radical scavenging capacities. Finally, among all selected compounds, **8** displays the shorter DPPH value.

DMSO affects oxidative stress-induced cytotoxicity, inhibits methionine sulfoxide reductase A, and reduces cell viability and survival, depending on the concentration used (as reviewed in [35], and references herein). However, methanol only affects yeast growth, by reducing its growth rate, due to the inhibition of nutrients uptake, when used at more than 10% concentration [36]. Therefore, we select methanol (up to 0.1%) as solvent for compounds in our study of determination of in vivo antioxidant and oxidant-resistance properties of the selected polyphenols. This study was performed as previously described; BY4741 yeast cells were exposed to H_2_O_2_ as oxidative agent for 16h, and then cultures were challenged by different doses to ascertain changes in growth curve parameters and in yeast cell survivorship [16,30]. Growth ability was assessed with Bioscreen c, on which H_2_O_2_-stressed cells were challenged by each compound **8**, **15** and **17**, along with Resveratrol and vitamin C as control antioxidants, at three different doses (2, 20 and 40 μg). Plates also contained several types of controls (un-inoculated media, un-inoculated media with methanol, completely stressed cells in YPOxD and un-stressed cells cultured in YPD medium), all at least in triplicate. From the visual analysis of growth curve shape, tested compounds were enhancing growth recovery as compared with completely stressed cells (OxDinOxD), similarly as resveratrol or vitamin C, the two control antioxidants used (see an example in Figure S3). However, nearly none of the tested doses achieved a complete recovery similar to non-oxidative stressed cells (YPD (CTRL)). 

**Figure 4:**
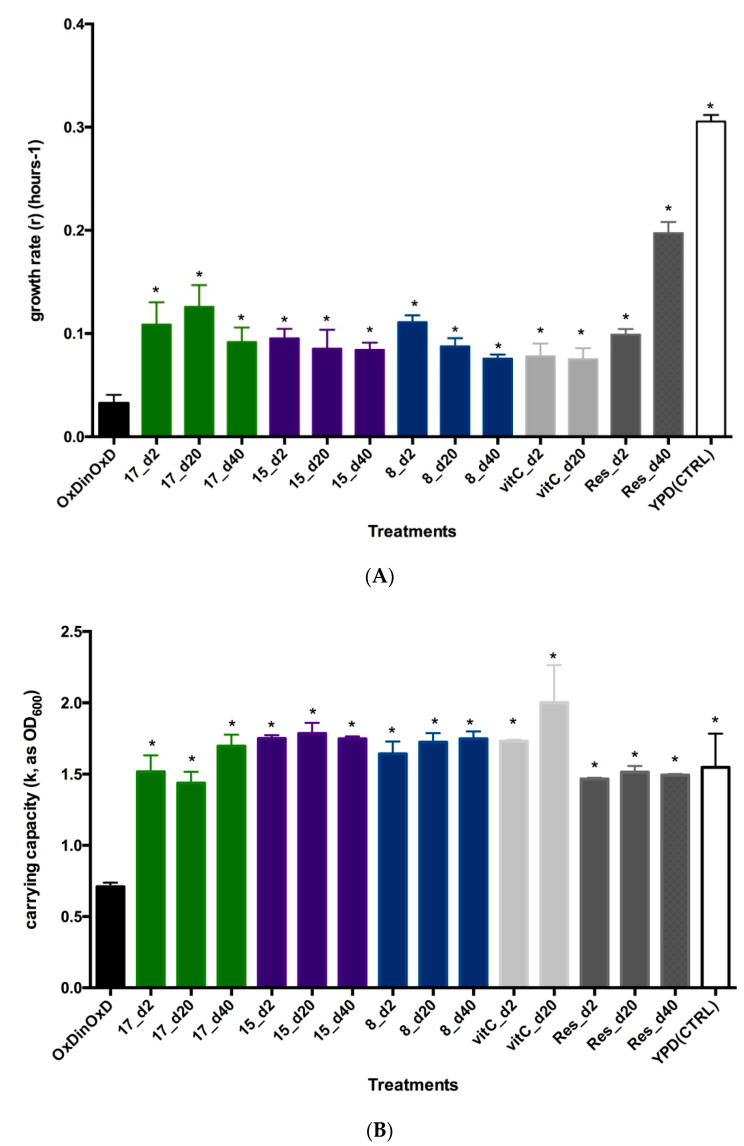
*Saccharomyces cerevisiae growth parameters comparisons.* (**A**) Comparison of growth rate (r) as mean ± SEM (h^−1^) of *S. cerevisiae* subjected to oxidative damage (grown in YPOxD medium) and challenged by different doses of compounds **17**, **15** and **8**, or by the antioxidants vitamin C (vitC) or resveratrol (Res). Comparisons have been made against completely stressed cells (OxDinOxD). To account for the oxidative damage, control lines (YPD(CTRL)) were also included, and consisted in *S. cerevisiae* cells grown in YPD (normal) medium, on which yeast cells can grow at maximum speed. Notice that oxidative stress reduces highly the growth rate, which is significantly recovered (*) after being challenged by compounds **17**, **15** or **8**, in a similar way as the control antioxidant resveratrol. (**B**) Comparison of carrying capacity (k) as mean ± SEM, after oxidative stress in *S. cerevisiae*. As above, yeast cultures responded positively recovering carrying capacity after being challenged by different doses of compounds **17**, **15** and **8**. In this case, carrying capacity was similarly recovered to unstressed yeast cells (YPD(CTRL)). Asterisks denote statistical significance with alpha 0.05.

To ascertain if this observed recovery of growth curve shape is statistically significant, two growth parameters were assessed: growth rate (r) and carrying capacity (k). Optical values (OD) were analyzed with Growthcurver R package, and obtained parameters r and k were compared with yeast cells under complete oxidative stress (OxDinOxD) or with completely unstressed cells (YPD (CTRL)). Growth rate (r) was significantly higher than the values obtained for completely stressed cells, indicating a recovery of biological functions in the yeast, but not the complete return to normality. As the unstressed yeasts, all tested compounds-treated yeast cells showed a highly significant higher growth rate (r = 0.3056 ± 0.0063 h^−1^; *F*(_22,46_) = 31.43; *p* < 0.0001). The comparison of growth rate (r) between treatments (Figure 4A) indicates that apparently the best performing antioxidant is compound **17** at each single dose tested, whereas compounds **15** and **8**, showed a dose-related antioxidant effect, with the best growth rate values at a 2-μg dose. None of the tested compounds nor control antioxidants, vitamin C or resveratrol, recover completely growth rate, as mean values were significantly different from unstressed yeast (YPD(CTRL)). This recovery of growth rate is similar to that obtained with other synthetic [16] or food-derived phenolic compounds like onion skin extracts or cocoa polyphenols [30,37] or elements like selenium [38]. When comparing the carrying capacity (k) (Figure 4B), all three compounds at each single dose tested were able to recover the carrying capacity of yeast cells similarly as control antioxidants, and even to the same levels of unstressed yeast cells. In this case, compound **15**, at a low 2 μg dose, seems slightly better than the other two polyphenols and resveratrol, and comparable to vitamin C. In general, measured growth rate and carrying capacity are quite similar for the three new polyphenols. Therefore, we validate the *S. cerevisiae* as a good model for measuring antioxidant activity in vivo. 

**Figure 5:**
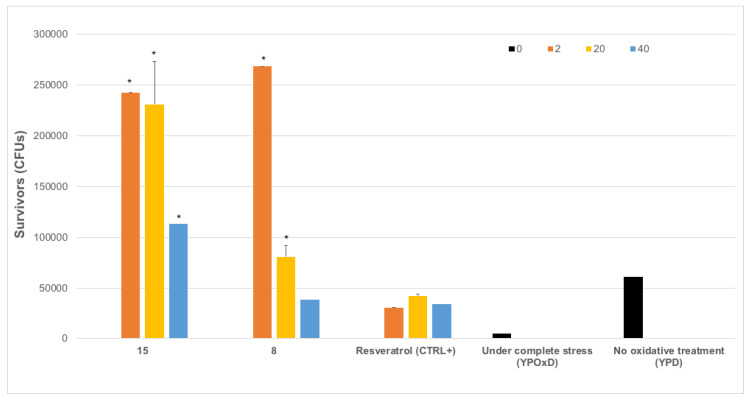
*Saccharomyces cerevisiae* survivors (mean CFUs (colony forming units) ± SEM) to oxidative stress after treatment with compounds **15** and **8**. Yeast cells were subjected to oxidative stress by growing in YPOxD medium, after 16h, cells were subjected to treatment with different doses (2, 10, 20 or 40 μg, check color code in legend) in triplicate, and after 60 h of growth monitorization by OD600 measurement in Bioscreen c, cells were serially diluted in 10% glycerol and plated in YPD medium plates, to determine number of CFUs per ml. Stressed yeasts without antioxidant treatment (under complete stress (YPOxD)), non-stressed yeasts (no oxidative treatment (YPD)) and stressed yeasts treated with resveratrol (resveratrol (CTRL+) were also included. Statistical significance (F-test with *p*-value lower than 0.05) was assessed first between YPOxD and YPD yeast survivors; and then between YPOxD stressed and antioxidant-treated, followed by comparison between control antioxidant (Resveratrol) and tested compounds at each dose. Asterisk indicates statistically significant comparisons (alpha = 0.05).

This carrying capacity also reflects the ability of yeast cells to respond to oxidative damage by producing viable offspring or survivors, which sometimes is called stress tolerance assays. These studies represent the capacity of the cells to counteract and resist the oxidative damage, representing cell survival [30,31,36,38,39,40,41,42]. Even showing statistical differences between applied doses, and being significantly different from completely stressed yeasts (OxDinOxD) and unstressed ones (YPD), our results indicate that tested compounds enhance biological functions enabling the complete recovery of cell biochemistry and metabolism Indeed, tested compounds counteract the oxidative damage induced by the used H_2_O_2_ medium and increase significantly the oxidative protection rate when compared to resveratrol (Figure 5). Considering how this effect is possible, we do not yet know about the exact molecular mechanisms behind this antioxidant activity. 

The obtained data supported the recovery of growth rate, whole metabolic capability and reproductive physiology by the selected synthetic polyphenols. However, the data also indicate that *S. cerevisiae* endogenous cellular antioxidative defense systems are enhanced at a similar or even better rate than those measured for well-known antioxidants resveratrol and vitamin C (Figure 5) [34,38].

## 4. Final Remarks and Conclusions

Polyphenols are widely distributed in nature (tea, coffee, chocolate, fruits, legumes, etc.), they function as antioxidants and protect us from free radical damage in several chronic diseases (cancer, diabetes, cardiovascular and neurodegenerative diseases). Among different applications, such as oil, food and cosmetic preservatives, synthetic polyphenols could also be of interest in the search for new bioactive agents. Following our previous work on antioxidant triazolyl polyphenols, and inspired by the OH substitutions in natural products, we designed a small collection of amide and urea polyhydroxyphenyl analogues. Synthetically more accessible than the triazolyl derivatives, compounds **1**–**18** were first analyzed by their antioxidant capacity in ORAC assays, with most compounds comparing favorably to resveratrol (6.6–27.6 TE versus 8.1 TE). Measurement of the aqueous stability allowed the identification of polyphenol derivatives prone to oxidization to the corresponding quinones, which were removed from further characterization to avoid misinterpretations. The most stable polyphenols (**3**, **7**, **8**, **15** and **17**) were selected for further characterization, using ABTS and DPPH experiments, where most derivatives showed comparable and even higher radical scavenging abilities than the natural product resveratrol, except for **15**. Selected compounds were further evaluated in different biological systems, namely endothelial cells, whole aorta and/or yeast. Compounds **3** and **7** were able to protect cultured human microvascular endothelial cells against oxidative stress promoted by Angiotensin II, decreasing the levels of NADPH activity. In addition, these polyphenols and urea **17** inhibited the effects of IL-1β-induced NADPH oxidase activity ex vivo, at 1nM concentration or higher, confirming their beneficial effects in oxidative stress models at the vascular level. To have in vivo evidence supporting the in vitro-based antioxidant activity of our compounds, a *Saccharomyces cerevisiae* model organism was used to investigate the effect of selected synthetic polyphenols with different in vitro profiles (**8**, **15** and **17**). All investigated amide and urea derivatives displayed similar in vivo antioxidant properties in this model, with recovery of growth rate and of whole metabolic capability similar or even better than model antioxidants (resveratrol and vitamin C). Polyphenol **17** is the most balanced compound in the series, because of the good in vitro, ex vivo and in vivo antioxidant properties, and its low toxicity. The synthetic polyphenols described here could have future applications in the treatment of aging and many clinical conditions characterized by high oxidative–stress levels, such as cancer, cardiovascular diseases, chronic obstructive pulmonary disease, chronic kidney illness, and different neurodegenerative processes. Confirmation of other therapeutic uses will require additional experiments on different models.

## Figures and Tables

**Table 1:**
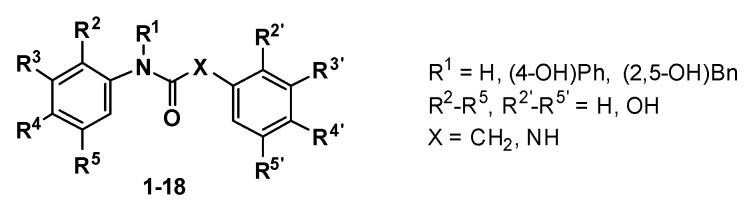
Structures and yield of synthetic polyhydroxy amides (**1** to **10**), and ureas (**11** to **18**) prepared.

Compound	R1	R2	R3	R4	R5	R2′	R3′	R4′	R5′	X	Yield (%)
1	H	OH	H	OH	H	OH	H	H	OH	CH_2_	41
2	H	OH	H	H	OH	OH	H	OH	H	CH_2_	44
3	H	H	H	OH	H	OH	H	H	OH	CH_2_	79
4	H	OH	H	H	OH	OH	H	H	OH	CH_2_	79
5	H	OH	H	OH	H	OH	H	OH	H	CH2	94
6	H	H	OH	OH	H	OH	H	H	OH	CH_2_	56
7	H	H	H	OH	H	H	OH	OH	H	CH_2_	75
8	H	H	H	OH	H	OH	H	OH	H	CH_2_	80
9	(4-OH)Ph	H	H	OH	H	OH	H	H	OH	CH_2_	72
10	(2,5-OH)Bn	H	H	OH	H	OH	H	H	OH	CH_2_	81
11	H	OH	H	OH	H	OH	H	H	OH	NH	95
12	H	OH	H	H	OH	H	H	OH	H	NH	56
13	H	OH	H	H	OH	H	OH	OH	H	NH	68
14	H	H	OH	OH	H	H	H	OH	H	NH	60
15	H	H	H	OH	H	H	H	OH	H	NH	84
16	H	OH	H	H	OH	OH	H	H	OH	NH	20
17	(4-OH)Ph	H	H	OH	H	H	H	OH	H	NH	68
18	(2,5-OH)Bn	H	H	OH	H	OH	H	H	OH	NH	10

**Table 2:**
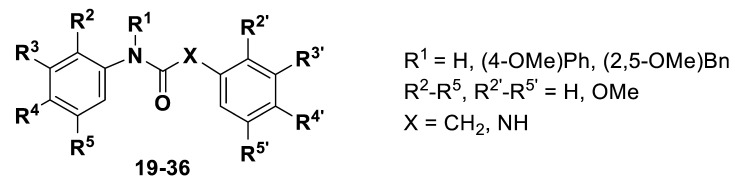
Structures and yield of OMe precursors prepared, amides **19** to **28** and ureas **29** to **36**.

Compound	R^1^	R^2^	R^3^	R^4^	R^5^	R^2′^	R^3′^	R^4′^	R^5′^	X	Yield (%)
19	H	OMe	H	OMe	H	OMe	H	H	OMe	CH_2_	81
20	H	OMe	H	H	OMe	OMe	H	OMe	H	CH_2_	77
21	H	H	H	OMe	H	OMe	H	H	OMe	CH_2_	50
22	H	OMe	H	H	OMe	OMe	H	H	OMe	CH_2_	65
23	H	OMe	H	OMe	H	OMe	H	OMe	H	CH_2_	74
24	H	H	OMe	OMe	H	OMe	H	H	OMe	CH_2_	73
25	H	H	H	OMe	H	H	OMe	OMe	H	CH_2_	81
26	H	H	H	OMe	H	OMe	H	OMe	H	CH_2_	76
27	(4-OMe)Ph	H	H	OMe	H	OMe	H	H	OMe	CH_2_	28
28	(2,5-OMe)Bn	H	H	OMe	H	OMe	H	H	OMe	CH_2_	74
29	H	OMe	H	OMe	H	OMe	H	H	OMe	NH	55
30	H	OMe	H	H	OMe	H	H	OMe	H	NH	85
31	H	OMe	H	H	OMe	H	OMe	OMe	H	NH	69
32	H	H	OMe	OMe	H	H	H	OMe	H	NH	42
33	H	H	H	OMe	H	H	H	OMe	H	NH	80
34	H	OMe	H	H	OMe	OMe	H	H	OMe	NH	84
35	(4-OMe)Ph	H	H	OMe	H	H	H	OMe	H	NH	63
36	(2,5-OMe)Bn	H	H	OMe	H	OMe	H	H	OMe	NH	17

**Table 3:** Antioxidant activity (oxygen radical absorbance capacity (ORAC)) and chemical stability of compounds **1** to **18**.

Compound	Chemical Structure	ORAC ^a^	Aqueous Stability ^b^(%)
1	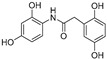	11.0 ± 0.4	2
2	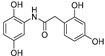	8.1 ± 0.1	-
3	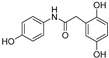	19.2 ± 0.2	77
4	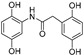	14.4 ± 0.2	0
5	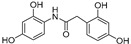	5.2 ± 0.4	-
6	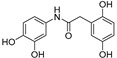	9.4 ± 0.4	0
7	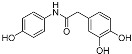	19.2 ± 0.4	90
8	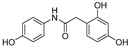	19.3 ± 0.5	98
9	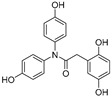	29.5 ± 0.5	0
10	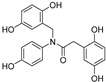	27.6 ± 0.5	0
11	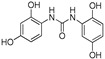	6.6 ± 0.1	-
12	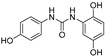	12.5 ± 0.7	0
13	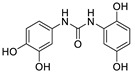	7.8 ± 0.3	-
14	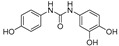	9.6 ± 0.1	0
15	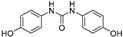	8.9 ± 0.4	90
16	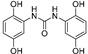	11.9 ± 0.4	0
17	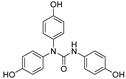	19.4 ± 0.7	93
18	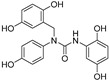	15.3 ± 0.7	0
Resveratrol	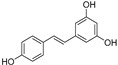	8.1 ± 1.17	-

^a^ µmol of trolox/µmol of pure compound. ^b^ Percentage of remaining pure compound (measured by HPLC) after two months at room temperature in aqueous solution (+20% acetonitrile).

**Table 4:** Antioxidant profile of selected stable compounds.

Comp	Chemical Structure	ORAC ^a^	ABTS ^a^	DPPH PSA IC50 (µM) ^b^	
				10 min	2 h
3	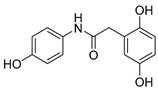	19.2 ± 0.2	3.27 ± 0.1	8.30	6.77
7	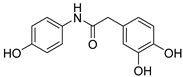	19.2 ± 0.	8.12 ± 0.09	24.10	23.30
8	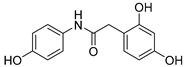	19.3 ± 0.5	5.00 ± 0.09	150.90	64.20
15	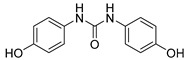	8.9 ± 0.4	1.17 ± 0.01	ND ^c^	ND ^c^
17	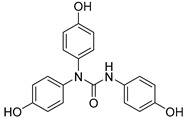	19.4 ± 0.7	3.57 ± 0.00	17.92	9.60
Resveratrol	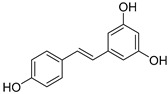	8.08 ± 1.2	2.13 ± 0.02	66.22	19.48

^a^ µmol of trolox/µmol of pure compound. ^b^ The half-maximal inhibitory concentration (IC_50_) values for the α,α-diphenyl-β-picrylhydrazyl (DPPH) assay correspond to the amount of compound needed to reach 50% of radical scavenging activity (RSA) after 10 min and 2 h of addition. ^c^ ND: not determined.

## Supplementary Materials

The following are available online at https://www.mdpi.com/2076-3921/9/9/787/s1, Chemical description of MeO analogues, Figure S1: HPLC-MS study of aqueous stability of compounds in aqueous solution, Table S1: Antioxidant evaluation of several natural products, Table S2: Theoretical Log P values and aqueous thermodynamic solubility measured for selected compounds, Table S3: Variations with sample concentration and time, Figure S2: Cytotoxicity, IC_50_ values for selected compounds and non-linear fits, Figure S3: In vivo growth ability of selected compounds in yeast.
