# Supplementary material for: Characterization of Novel Synthetic Polyphenols: Validation of Antioxidant and Vasculoprotective Activities
Source: Antioxidants (Basel). 2020 Aug 25;9(9):787. doi: 10.3390/antiox9090787 (PMC7555119; doi:10.3390/antiox9090787)
Supplement: Supplementary file 1 [file antioxidants-09-00787-s001.pdf]

Supplementary Content

Characterization of Novel Synthetic Polyphenols: Validation of Antioxidant and Vasculoprotective Activities

Maria J Perez de Vega [1], Silvia Moreno-Fernandez [2], Gloria Pontes [3,6], María González-Amor[4,7], Blanca Vázquez [3,8], Beatriz Sabater-Munoz [5], Ana M. Briones [4,7], Maria R. Aguilar [3,8], Marta Miguel[2] and Rosario Gonzalez-Muniz [1,*]

[1]*Instituto de Química Médica, IQM-CSIC, Juan de la Cierva 3, 28006 Madrid, Spain; e-mail@e-mail.com*
[2]*Instituto de Investigación en Ciencias de la Alimentación (CSIC-UAM, CEI+UAM), C/ Nicolás Cabrera 9, 28049 Madrid, Spain*
[3]*Instituto de Ciencia y Tecnología de Polímeros, IQM-CSIC, Juan de la Cierva 3, 28006 Madrid, Spain*
[4]*Facultad de Medicina, Departamento de Farmacología, Universidad Autónoma de Madrid, Arzobispo Morcillo 4, 28029 Madrid. Instituto de Investigaciones Biomédicas Hospital La Paz. Spain,*
[5]*Instituto de Biología Molecular y Celular de Plantas (IBMCP, CSIC-UPV), Ingeniero Fausto Elio, 46022, Valencia, Spain*
[6]*Alodia Farmacéutica SL, Santiago Grisolía 2 D130/L145, Madrid, Spain.*
[7]*Centro de Investigación Biomédica en Red de Enfermedades Cardiovasculares (CIBERCV), Spain.*
[8]*Networking Biomedical Research Centre in Bioengineering, Biomaterials and Nanomedicine, CIBER-BBN, Madrid, Spain.*

1) Chemistry. S2

2) Chemical stability of compounds in aqueous solution S11

3) Antioxidant properties for several natural products S13

4) Log P and aqueous solubility of prepared compounds S14

5) Results for DPPH antioxidant capacity assay S15

6) Cell viability assay S16

7) *In vivo* growth ability induced by selected compounds in yeast S17

1) Chemistry.

General Information. All reagents were of commercial quality. Solvents were dried and purified by standard methods. Analytical TLC was performed on aluminum sheets coated with a 0.2 mm layer of silica gel 60 F254. Silica gel 60 (230-400 mesh) was used for flash chromatography. Unless indicated, all compounds were isolated in a purity ≥ 95% (HPLC data). Analytical HPLC-MS was performed on a Waters 2695 equipment coupled to a single quadrupole ESI-MS (Waters Micromass ZQ 2000) using a reverse-phase SunFire™ C18 4.6 x 50 mm column (3.5 μm) at a flow rate of 1 mL/min and by using a diodo array UV detector. Mixtures of CH_3CN + 0.08% formic acid (solvent A) and H_2O + 0.1% formic acid (solvent B) were used as mobile phase (gradients of 2 or 15 to 95% of A in B in 5 or 10 min, as indicated in each case. NMR spectra were recorded on a Varian-INOVA 300, a Bruker-AVANCE 300, a Varian-MERCURY 400, a Varian-INOVA 400 or a Varian System 500 spectrometer, operating at 300, 400 or 500 MHz for ^1H and at 75, 100, and 125 MHz for ^{13}C recording. To confirm the NMR peak assignments, COSY and HSQC experiments were performed when necessary. Melting points were determined on a Mettler MP70 apparatus and are uncorrected.

Structures of compounds **15**, **33** and **34** were confirmed by comparison of their physical data with those reported in the literature.

General procedure for the synthesis of methoxy-substituted amides 19 to 28.

To a solution of the corresponding OMe-substituted aniline (1.52 mmol) in CH_2Cl_2 (15 mL), the corresponding phenylacetic acid (1.52 mmol), HOBt (0.22 g, 1.68 mmoles), DIEA (0.198 g, 1.52 mmoles) were added. The solution was cooled in an ice bath, and EDC·HCl (0.35 g, 1.83 mmoles) was added. After two hours the ice bath was removed and the mixture was stirred at room temperature for 12 h. The reaction mixture was

successively washed with citric acid 10 %, $NaCO_3H$ 10%, water and brine, dried over $MgSO_4$ and the solvent removed under vacuum to dryness. The resulting residue was crystallized or chromatographed, as indicated in each case, leading to the corresponding polymetoxylated amides.

N-(2,4-Dimethoxyphenyl)-2-(2',5'-dimethoxyphenyl)acetamide (19)

Grey crystalline solid, 81% yield, m.p.: 103-105ºC (MeOH). HPLC: t_R = 8.93 min (10 min gradient: 15 to 95% of A in B). ^{1}H-NMR (400 MHz, DMSO-d_6) δ: 3.60 (s, 2H, CH_2), 3.70 (s, 3H, OMe), 3.72 (s, 3H, OMe), 3.77 (s, 3H, OMe), 3.80 (s, 3H, OMe), 6.46 (dd, 1H, J = 8.8, 2.6 Hz, 4'-H), 6.60 (d, 1H, J = 2.6 Hz, 6'-H), 6.81 (dd, 1H, J = 8.9, 3.0 Hz, 5-H), 6.87 (d, 1H, J = 3.0 Hz, 3-H), 6.93 (d, 1H, J = 8.9 Hz, 6-H), 7.80 (d, 1H, J = 8.8 Hz, 3'-H), 8.84 (s, 1H, NH) ppm. ^{13}C-NMR (100 MHz, DMSO-d_6) δ: 38.3 (CH_2), 55.3 (OMe), 55.4 (OMe), 55.8 (OMe), 55.9 (OMe), 98.7 (C3), 104.0 (C5), 111.7 (C3'), 112.3 (C4'), 116.9 (C6'), 120.7 (C6), 122.1 (C1), 125.1 (C1'), 150.4 (C), 151.1 (C), 153.0 (C), 156.3 (C), 168.4 (CO) ppm. MS (ESI$^+$): m/z 332.4 $(M+H)^+$, 354.5 $(M+Na)^+$.

2-(2',4'-Dimethoxyphenyl)-N-(2,5-dimethoxyphenyl)acetamide (20)

Grey crystalline solid, 77% yield, m.p.: 117-119ºC (MeOH). HPLC: t_R = 9.48 min (10 min gradient: 15 to 95% of A in B). ^{1}H-NMR (300 MHz, DMSO-d_6) δ: 3.58 (s, 2H, CH_2), 3.65 (s, 3H, OMe), 3.76 (s, 6H, OMe), 3.82 (s, 3H, OMe), 6.51 (dd, 1H, J = 8.3, 2.4 Hz, 4'-H), 6.57 (dd, 1H, J = 8.9, 3.0 Hz, 5-H), 6.60 (d, 1H, J = 2.4 Hz, 6'-H), 6.92 (d, 1H, J = 8.9 Hz, 6-H), 7.14 (d, 1H, J = 8.3 Hz, 3'-H), 7.78 (d, 1H, J = 3.0 Hz, 3-H), 8.81 (s, 1H, NH) ppm. ^{13}C-NMR: (75 MHz, DMSO-d_6) δ: 38.2 (CH_2), 55.2 (OMe), 55.3 (OMe), 55.6 (OMe), 56.3 (OMe), 98.4 (C3), 104.8 (C5), 106.6 (C1), 107.3 (C6'), 111.6 (C4'), 115.8 (C5'), 128.4 (C1'), 131.3 (C6), 142.4 (C), 153.0 (C), 157.6 (C), 159.8 (C), 169.4 (CO) ppm. MS (ESI$^+$): m/z 332.4 $(M+H)^+$, 354.5 $(M+Na)^+$.

2-(2',5'-Dimethoxyphenyl)-N-(4-methoxyphenyl)acetamide (21)

White prisms, 50% yield, m.p.: 145-146ºC (MeOH). HPLC: t_R = 4.29 min (5 min gradient: 15 to 95% of A in B). 1H-NMR (300 MHz, CDCl3) δ: 3.70 (s, 2H, CH2), 3.79 (s, 3H, OMe), 3.80 (s, 3H, OMe), 3.91 (s, 3H, OMe), 6.85 (m, 3H, 3,4, 4'-H), 6.91 (m, 2H, 3-H, 5-H), 7.37 (d, 2H, J = 8.6 Hz, 2-H, 6-H), 7.76 (br s, 1H, NH) ppm. 13C-NMR: (75 MHz, CDCl3) δ: 40.2 (CH2), 55.5 (OMe), 55.8 (OMe), 56.2 (OMe), 112.1 (C2'), 113.6 (C4'), 114.1 (C3, C5), 117.1 (C6'), 121.6 (C2, C6), 124.2 (C1'), 131.4 (C1), 151.2 (C), 154.0 (C), 156.3 (C), 169.6 (CO) ppm. MS (ESI+): m/z 302.28 $(M+H)^+$.

_N_-(2,5-Dimethoxyphenyl)-2-(2',5'-dimethoxyphenyl)acetamide (22)

White prisms, 65% yield, m.p.: 109-111ºC (MeOH). HPLC: t_R = 4.82 min (5 min gradient: 15 to 95% of A in B). ^{1}H-NMR (300 MHz, $CDCl_3$) δ: 3.74 (s, 2H, CH_2), 3.79 (s, 6H, OMe), 3.80 (s, 3H, OMe), 3.92 (s, 3H, OMe), 6.55 (dd, 1H, J = 8.3, 3.1 Hz, 4-H), 6.77 (d, 1H, J = 8.9 Hz, 3'-H), 6.88 (m, 3H, 3,4',6'-H), 8.15 (d, 1H, J = 3.1 Hz, 6-H), 8.55 (br s, 1H, NH) ppm. ^{13}C-NMR: (75 MHz, $CDCl_3$) δ: 40.8 (CH_2), 55.9 (OMe), 56.9 (OMe), 56.5 (OMe), 105.8 (C6), 108.4 (C3), 111.0 (C4), 111.5 (C3'), 113.6 (C4'), 117.0 (C6'), 124.4 (C1'), 128.9 (C1), 142.1 (C), 151.1 (C), 153.9 (C), 154.0 (C), 169.3 (CO) ppm. MS (ESI^+): *m/z* 332.35 $(M+H)^+$.

_N_-(2,4-Dimethoxyphenyl)-2-(2',4'-dimethoxyphenyl)acetamide (23)

Brownish crystalline solid, purified by column chromatography EtOAc-Hex (gradient from 1:3 to 2:1). 74% yield, m.p.: 125-126ºC (MeOH). HPLC: t_R = 4.70 min (5 min gradient: 15 to 95% of A in B). ^{1}H-NMR (300 MHz, $CDCl_3$) δ: 3.62 (s, 2H, CH_2), 3.76 (s, 6H, OMe), 3.80 (s, 3H, OMe), 3.87 (s, 3H, OMe), 6.35-6.56 (m, 4H, 3,5-,3',5'-H), 7.24 (d, 1H, J = 7.9 Hz, 6'-H), 8.11 (br s, 1H, NH), 8.22 (d, 1H, J = 8.3 Hz, 6-H) ppm. ^{13}C-NMR: (75 MHz, $CDCl_3$) δ: 39.6 (CH_2), 55.5 (OMe), 55.5 (OMe), 55.6 (OMe), 55.8 (OMe), 98.7 (C-3), 98.8 (C3'), 103.8 (C5'), 104.7 (C5), 116.2 (C6), 120.4 (C6'), 121.8 (C1'), 131.7 (C1), 149.2 (C), 156.1 (C), 158.0 (C), 160.5 (C), 169.5 (CO) ppm. MS (ESI^+): *m/z* 331.93 $(M+H)^+$.

2-(2',5'-Dimethoxyphenyl)-*N*-(3,4-dimethoxyphenyl)acetamide (24)

White prisms, 73.5% yield, m.p.: 140-142ºC (MeOH). HPLC: t_R = 4.09 min (5 min gradient: 15 to 95% of A in B) ^{1}H-NMR (300 MHz, $CDCl_3$) δ: 3.71 (s, 2H, CH_2), 3.76 (s, 3H, OMe), 3.82 (s, 3H, OMe), 3.84 (s, 3H, OMe), 3.87 (s, 3H, OMe), 6.70-7.34 (m, 6H, Ar-H), 7.71 (br s, 1H, NH) ppm. ^{13}C-NMR: (75 MHz, $CDCl_3$) δ: 40.3 (CH_2), 55.8 (OMe), 56.0 (OMe), 56.2 (OMe), 105.0 (C2), 111.4 (C5), 111.6 (C3'), 112.1 (C4'), 113.6 (C6), 117.2 (C6'), 124.5 (C1'), 132.0 (C-1), 145.8 (C), 149.1(C), 151.2 (C), 154.1 (C), 169.2 (CO) ppm. MS (ESI$^+$): *m/z* 332.28 $(M+H)^+$.

2-(3',4'-Dimethoxyphenyl)-*N*-(4-methoxyphenyl)acetamide (25)

White prisms. 81%, m.p.: 143-146ºC (MeOH). HPLC: t_R = 3.90 min (5 min gradient: 15 to 95% of A in B) ^{1}H-NMR (400 MHz, $CDCl_3$) δ: 3.66 (s, 2H, CH_2), 3.77 (s, 3H, OMe), 3.89 (s, 3H, OMe), 3.90 (s, 3H, OMe), 6.28 (d, 2H, J = 8.9 Hz, 3-H, 5-H), 6.83-6.90 (m, 3H, 2'-H, 5'-H, 6'-H), 7.04 (bs, 1H, NH), 7.31 (d, 2H, J = 8.9 Hz, 2-H, 6-H) ppm. ^{13}C-NMR: (75 MHz, $CDCl_3$) δ: 44.3 (CH_2), 55.5 (OMe), 56.0 (OMe), 111.8 (C2'), 112.7 (C5'), 114.2 (C3, C5), 121.8 (C2, C6, C6'), 127.2 (C1'), 130.9 (C1), 148.6 (C), 149.5(C), 156.6 (C), 169.4 (CO) ppm. MS (ESI$^+$): *m/z* 302.21 $(M+H)^+$.

2-(2',4'-Dimethoxyphenyl)-*N*-(4-methoxyphenyl)acetamide (26)

White prisms, 76% yield, m.p.: 126-128ºC, (MeOH). HPLC: t_R = 4.35 min (5 min gradient: 15 to 95% of A in B). ^{1}H-NMR (400 MHz, $CDCl_3$) δ: 3.62 (s, 2H, CH_2), 3.76 (s, 3H, OMe), 3.82 (s, 3H, OMe), 3.88 (s, 3H, OMe), 6.51 (m, 2H, 4'-H, 5'-H), 6.80 (d, 2H, J= 8.9 Hz, 3-H, 5-H), 7.19 (d, 1H, J = 8.0 Hz, 3'-H), 7.32 (d, 2H, J = 8.9 Hz, 2-H, 6-H), 7.43 (br s, 1H, NH) ppm. ^{13}C-NMR: (75 MHz, $CDCl_3$) δ: 39.2 (CH_2), 55.5 (OMe), 55.6 (OMe), 55.7 (OMe), 99.1 (C3'), 105.2 (C5'), 114.1 (C3, C5), 115.9 (C1'), 121.6 (C2, C6), 131.4 (C1), 131.8 (C6'), 156.3 (C), 158.2 (C), 160.7 (C), 169.7 (CO) ppm. MS (ESI$^+$): *m/z* 302.35 $(M+H)^+$.

2-(2',5'-Dimethoxyphenyl)-*N,N*-bis(4-methoxyphenyl)acetamide (27)

Colorless oil, 28% yield. HPLC: t_R = 7.62 min (10 min gradient: 15 to 95% of A in B).. ^{1}H-NMR (300 MHz, $CDCl_3$) δ: 3.42 (s, 2H, CH_2), 3.67 (s, 3H, OMe), 3.68 (s, 3H, OMe), 3.73 (s, 6H, OMe), 6.72-7.30 (m, 11H, Ar-H) ppm. ^{13}C-NMR: (75 MHz, $CDCl_3$) δ: 37.0 (CH_2), 55.6 (OMe), 55.7 (OMe), 55.9 (OMe), 56.0 (OMe), 11.3 (C-2'), 112.7 (C-4'), 114.2 (C-3, C-5), 114.8 (C3'',C5''), 116.8 (C6'), 125.7 (C1'), 127.4 (C2, C6), 129.8 (C2'',C6''), 136.4 (C-1', C1''), 151.7 (C), 153.6 (C),157.5 (C), 158.9 (C), 171.7 (CO) ppm. MS (ESI$^+$): *m/z* 408.39 $(M+H)^+$.

***N*-(2',5'-Dimethoxybenzyl)-2-(2'',5''-dimethoxyphenyl)-*N*-(4-methoxyphenyl) acetamide (28)**

White prisms, purified by column chromatography EtOAc-Hex (gradient from 1:4 to 1:3), 74% yield, m.p.: 127-129ºC (MeOH). HPLC: t_R = 8.02 min (10 min gradient: 2 to 95% of A in B). ^{1}H-NMR (300 MHz, $CDCl_3$) δ: 3.35 (s, 2H, CH_2), 3.58 (s, 3H, OMe), 3.65 (s, 3H, OMe), 3.67 (s, 3H, OMe), 3.68 (s, 3H, OMe), 3.73 (s, 3H, OMe), 6.73-6.86 (m, 6H, Ar-H), 6.91 (d, 2H, J = 9.0 Hz, 3-H, 5-H), 7.13 (d, 2H, J = 8.9 Hz, 2-H, 6-H) ppm. ^{13}C-NMR: (75 MHz, $CDCl_3$) δ: 36.0 (CH_2), 47.7 (*N*-CH_2), 55.5 (OMe), 55.8 (OMe), 55.85 (OMe), 55.9 (OMe), 56.0 (OMe), 111.2 (C2''), 111.6 (C2'), 112.5 (C4''), 112.8 (C4'), 114.2 (C3, C5), 115.9 (C6''), 116.8 (C6'), 126.0 (C1'), 127.2 (C1''), 129.5 (C2, C6), 135.8 (C-1), 151.6 (C), 151.8 (C), 153.5 (C), 153.7 (C), 158.8 (C), 171.7 (CO) ppm. MS (ESI$^+$): *m/z* 452.33 $(M+H)^+$.

General procedure for the synthesis of methoxy-substituted ureas 29 to 36.

To a solution of the conveniently substituted aniline (1,2 mmoles) in dry THF (20 mL), the corresponding phenylisocyanate (1.0 mmoles) was added. The reaction was stirred for 15 h at room temperature, and then, the solvent was removed and the residue treated with Cl_2CH_2. The organic phase was washed with water and brine, dry over anhydrous $MgSO_4$

and the solvent removed to dryness. The resulting residue was purified by crystallization or flash chromatography as indicated in each case, to give the polymethoxylated ureas.

***N*-(2,4-Dimethoxyphenyl)-*N*'-(2',5'-dimethoxyphenyl)urea (29)**

White lyophilized solid, purified by column chromatography EtOAc-Hex (gradient from 1:3 to 2:1), 55% yield, m.p.: 150-152ºC (MeOH). HPLC: t_R = 8.96 min (10 min gradient: 15 to 95% of A in B). ^{1}H-NMR (400 MHz, DMSO-d_6) δ: 3.67 (s, 3H, OMe), 3.73 (s, 3H, OMe), 3.80 (s, 3H, OMe), 3.84 (s, 3H, OMe), 6.45-6.47 (m, 2H, 4'-H, 5-H), 6.60 (d, 1H, J = 2.7 Hz, 3-H), 6.89 (d, 1H, J = 8.8 Hz, 3'-H), 7.83 (d, 1H, J = 3.0 Hz, 6'-H), 7.85 (d, 1H, J = 8.8 Hz, 6-H), 8.70 (s, 1H, NH), 8.73 (s, 1H, NH) ppm. ^{13}C-NMR (100 MHz, DMSO-d_6) δ: 55.2 (OMe), 55.3 (OMe), 55.7 (OMe), 56.3 (OMe), 98.7 (C3), 104.0 (C6'), 105.1 (C5), 105.5 (C4'), 111.4 (C3'), 120.9 (C1), 121.7 (C6), 130.0. (C1'), 142.1 (C), 149.9 (CO), 152.8 (C), 153.3 (C), 155.2 (C) ppm. MS (ESI$^+$): 333.3 (M+H)$^+$.

***N*-(2,5-Dimethoxyphenyl)-*N*'-(4'-methoxyphenyl)urea (30)**

White needles, 85% yield, m.p.: 162-164ºC (Cl_2CH_2) HPLC: t_R = 8.62 min (10 min gradient: 15 to 95% of A in B). ^{1}H-NMR (300 MHz, DMSO-d_6) δ: 3.68 (s, 3H, OMe), 3.71 (s, 3H, OMe), 3.81 (s, 3H, OMe), 6.46 (dd, 1H, J = 8.8, 3.0 Hz, 4-H), 6.86-6.91 (m, 3H, 3'-H, 5'-H, 3-H), 7.37 (d, 2H, J = 8.6 Hz, 2'-H, 6'-H), 7.37 (d, 1H, J = 2.9 Hz, 6-H), 8.16 (s, 1H, NH), 9.16 (s, 1H, NH) ppm. ^{13}C-NMR (75 MHz, DMSO-d_6) δ: 55.2 (OMe), 55.3 (OMe), 56.3 (OMe), 105.1 (C6), 105.2 (C4), 111.4 (C3), 114.1 (C3', C5'), 119.8 (C2', C6'), 129.8 (C1), 132.8. (C1'), 141.8 (C),152.5 (CO), 153.4 (C), 154.5 (C) ppm. MS (ESI$^+$): 303.3 (M+H)$^+$.

***N*-(2,5-Dimethoxyphenyl)-*N*'-(3',4'-dimethoxyphenyl)urea (31)**

White needles, 69% yield, m.p.: 159-161ºC (Cl_2CH_2/MeOH). HPLC: t_R = 8.82 min (10 min gradient: 15 to 95% of A in B). ^{1}H-NMR (300 MHz, DMSO-d_6) δ: 3.68 (s, 3H, OMe), 3.73 (s, 3H, OMe), 3.80 (s, 3H, OMe), 3.84 (s, 3H, OMe), 6.44-6.49 (m, 2H, 4-H, 6'-H), 6.60

(d, 1H, J = 2.5 Hz, 2'-H), 6.89 (d, 1H, J = 8.9 Hz, 3-H), 7.84-7.88 (m, 2H, 6-H, 5'-H), 8.69 (s, 1H, NH), 8.72 (s, 1H, NH) ppm. ^{13}C-NMR (75 MHz, DMSO-d_6) δ: 55.2 (OMe), 55.3 (OMe), 55.7 (OMe), 56.3 (OMe), 98.8 (C2'), 104.1 (C6), 105.2 (C4), 105.5 (C5'), 111.5 (C3), 120.9 (C6), 121.7 (C1), 130.0. (C1'), 142.1 (C), 149.9 (C), 152.8 (C), 153.3 (CO), 155.2 (C) ppm. MS (ESI$^+$): 333.4 (M+H)$^+$.

***N*-(3,4-Dimethoxyphenyl)-*N*'-(4'-methoxyphenyl)urea (32)**

White flakes, 42% yield, m.p.: 185-187ºC (EtOH). HPLC: t_R = 8.35 min (10 min gradient: 15 to 95% of A in B). ^{1}H-NMR (300 MHz, DMSO-d_6) δ: 3.71 (s, 3H, OMe), 3.73 (s, 3H, OMe), 3.85 (s, 3H, OMe), 6.47 (dd, 1H, J = 8.7, 2.4 Hz, 6-H), 6.61 (d, 1H, J = 2.4 Hz, 2-H), 6.86 (d, 1H, J = 8.7 Hz, 3'-H, 5'-H), 7.34 (d, 1H, J = 8.6 Hz, 2'-H, 6'-H), 7.88 (s, 1H, NH), 7.93 (d, 1H, J = 8.8 Hz, 5-H), 8.95 (s, 1H, NH) ppm. ^{13}C-NMR (75 MHz, DMSO-d_6) δ: 55.2 (OMe), 55.3 (OMe), 55.8 (OMe), 98.8 (C2), 104.1 (C5), 114.0 (C3', C5'), 119.5 (C2', C6'), 119.6 (C6), 122.1 (C1), 133.1 (C1), 149.1 (C), 152.8 (C), 154.3 (CO), 154.8 (C) ppm. MS (ESI$^+$): 303.99 (M+H)$^+$.

***N,N*'-bis(4-Methoxyphenyl)urea (33)**

White prisms, 80% yield, m.p.: 237-239ºC (EtOH; m.p. Lit [1] = 236-238ºC). HPLC: t_R = 7.88 min (10 min gradient: 15 to 95% of A in B). ^{1}H-NMR (300 MHz, DMSO-d_6) δ: 3.71 (s, 6H, OMe), 6.86 (d, 4H, J = 8.8, 3-H, 5-H), 7.35 (d, 4H, J = 8.8 Hz, 2-H, 6-H), 8.36 (s, 1H, NH) ppm. ^{13}C-NMR (75 MHz, DMSO-d_6) δ: 55.1 (OMe), 113.9 (C3, C5), 119.9 (C2, C6), 132.9 (C1), 153.0 (CO), 154.3 (C4) ppm. MS (ESI$^+$): 273.10 (M+H)$^+$.

***N,N*'-bis(2,5-Dimethoxyphenyl)urea (34)**

White needles, 84% yield, m.p.: 207-209ºC (MeOH; m.p. Lit [2] = 209ºC). HPLC: t_R = 9.04 min (10 min gradient: 15 to 95% of A in B). ^{1}H-NMR (300 MHz, DMSO-d_6) δ: 3.69 (s, 6H, OMe), 3.80 (s, 6H, OMe), 6.49 (dd, 2H, J = 8.8, 3.0 Hz, 4-H), 6.90 (d, 2H, J = 8.9 Hz, 3-H), 7.84 (d, 2H, J = 2.9 Hz, 6-H), 8.96 (d, 2H, NH) ppm. ^{13}C-NMR (75 MHz,

DMSO-d_6) δ: 55.3 (OMe), 56.3 (OMe), 105.5 (C6), 106.0 (C4), 111.54 (C3), 129.7 (C1), 142.4 (C2), 152.6 (CO), 153.3 (C5) ppm. MS (ESI$^+$): 333.0 $(M+H)^+$.

***N,N*-bis(4-Methoxyphenyl)-*N*'-(4'-methoxyphenyl)urea (35)**

White solid, 63% yield, m.p.: 153-156ºC (MeOH). HPLC: t_R = 7.24 min (10 min gradient: 15 to 95% of A in B). ^{1}H-NMR (400 MHz, $CDCl_3$) δ: 3.77 (s, 3H, OMe), 3.81 (s, 6H, OMe), 6.31 (sa, 1H, NH), 6.81 (d, 2H, *J* = 9.0 Hz, 3'-H, 5'-H), 6.91 (d, 4H, *J* = 8.8 Hz, 3-H, 5-H), 7.25 (d, 2H, *J*= 9.0 Hz, 2'-H, 6'-H), 7.27 (d, 4H, *J* = 8.8 Hz, 2-H, 6-H) ppm. ^{13}C-NMR: (75 MHz, $CDCl_3$) δ: 55.6 (OMe), 114.2 (C3',C5'), 114.9 (C3,C5), 121.3 (C2',C6'), 128.8 (C2,C6), 131.9 (C), 135.5 (C), 154.4 (C), 155.8 (CO), 158.1(C) ppm. MS (ESI$^+$): *m/z* 379.05 $(M+H)^+$.

***N*-(2',5'-Dimethoxybenzyl)-N'-(2'',5''-dimethoxyphenyl)-*N*-(4-methoxyphenyl) urea (36)**

Following the general procedure, using 1.5 mmol of amine. White prisms, purified by column chromatography EtOAc-Hex (gradient from 1:3 to 3:1), 17% yield, m.p.: 91-94ºC (Cl_2CH_2/cyclohexane). HPLC: t_R = 7.33 min (10 min gradient: 30 to 95% of A in B). ^{1}H-NMR (400 MHz, DMSO-d_6) δ: 3.53 (s, 3H, OMe), 3.61 (s, 3H, OMe), 3.66 (s, 3H, OMe), 3.67 (s, 3H, OMe), 3.77 (s, 3H, OMe), 4.81 (s, 2H, *N*-CH_2), 6.46 (dd, 1H, *J* = 8.8, 3.0 Hz, 4''-H), 6.77 (dd, 1H, *J* = 8.9, 2.9 Hz, 4'-H), 6.78 (d, 1H, 3'-H), 6.84-6.86 (m, 2H, 3''-H, 6'-H), 7.00 (d, 2H, *J* = 8.8 Hz, 3-H, 5-H), 7.11 (s, 1H, NH), 7.26 (d, 2H, *J* = 8.8 Hz, 2-H, 6-H), 7.77 (d, 1H, *J* = 3.0 Hz, 6''-H) ppm. ^{13}C-NMR (100 MHz, DMSO-d_6) δ: 47.25 (CH_2), 55.2 (OMe), 55.3 (OMe), 55.4 (OMe), 55.7 (OMe), 56.5 (OMe), 104.6 (C6''), 105.6 (C4''), 111.6 (C4'), 111.7 (C3'), 112.0 (C3''), 114.9 (C6'), 115.0 (C3,5), 127.0 (C), 129,3 (C2, C6), 129.5. (C), 133.5 (C), 141.6 (C), 150.9 (C), 153.0 (CO), 153.4 (C), 153.7 (C), 158.5 (C) ppm. MS (ESI$^+$): 453.1$(M+H)^+$.

References

1. Iqbal, A. F. M. Iron Carbonyls in Organic Synthesis, I. Iron Pentacarbonyl Induced Decomposition and Transfer Hydrogenation of Aryl Azides to Substituted Ureas. *Helv. Chim. Acta* **1976**, *59* (2), 655–660. https://doi.org/10.1002/hlca.19760590231.

2. Zhou, S.; Yao, T.; Yi, J.; Li, D.; Xiong, J. A Simple and Efficient Synthesis of Diaryl Ureas with Reduction of the Intermediate Isocyanate by Triethylamine. *J. Chem. Res.* **2013**, *37* (5), 315–319. https://doi.org/10.3184/174751913X13663925002708.

2) Chemical stability of compounds in aqueous solution.

1

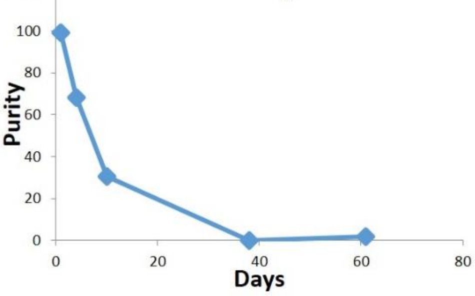

3

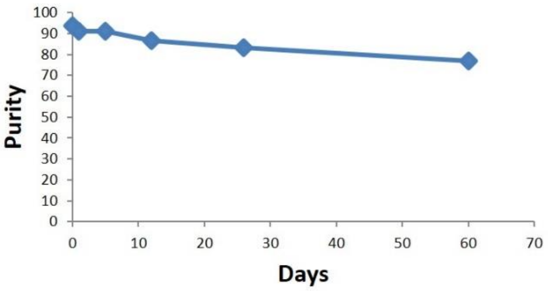

4

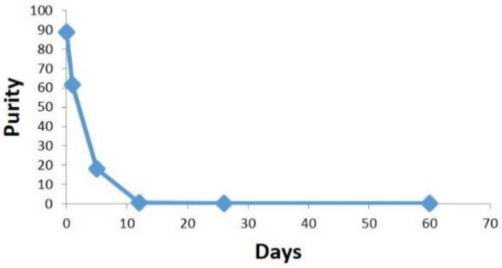

6

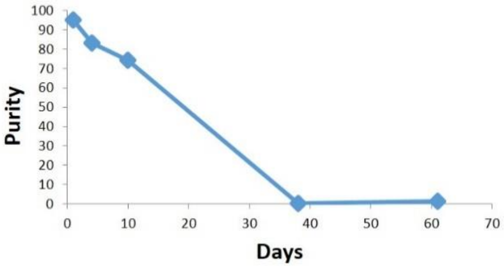

7

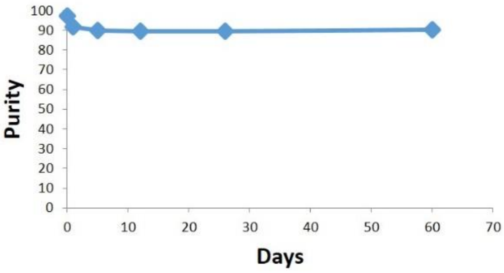

8

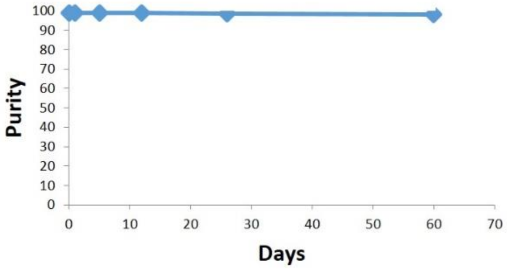

9

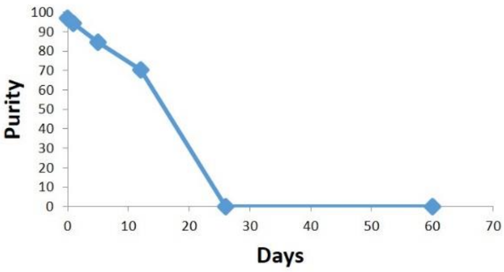

10

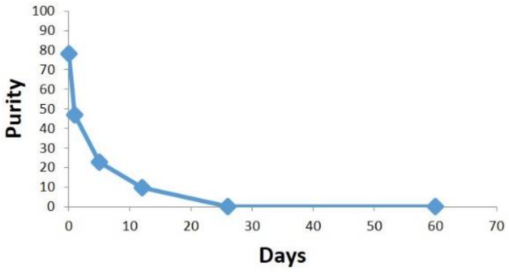

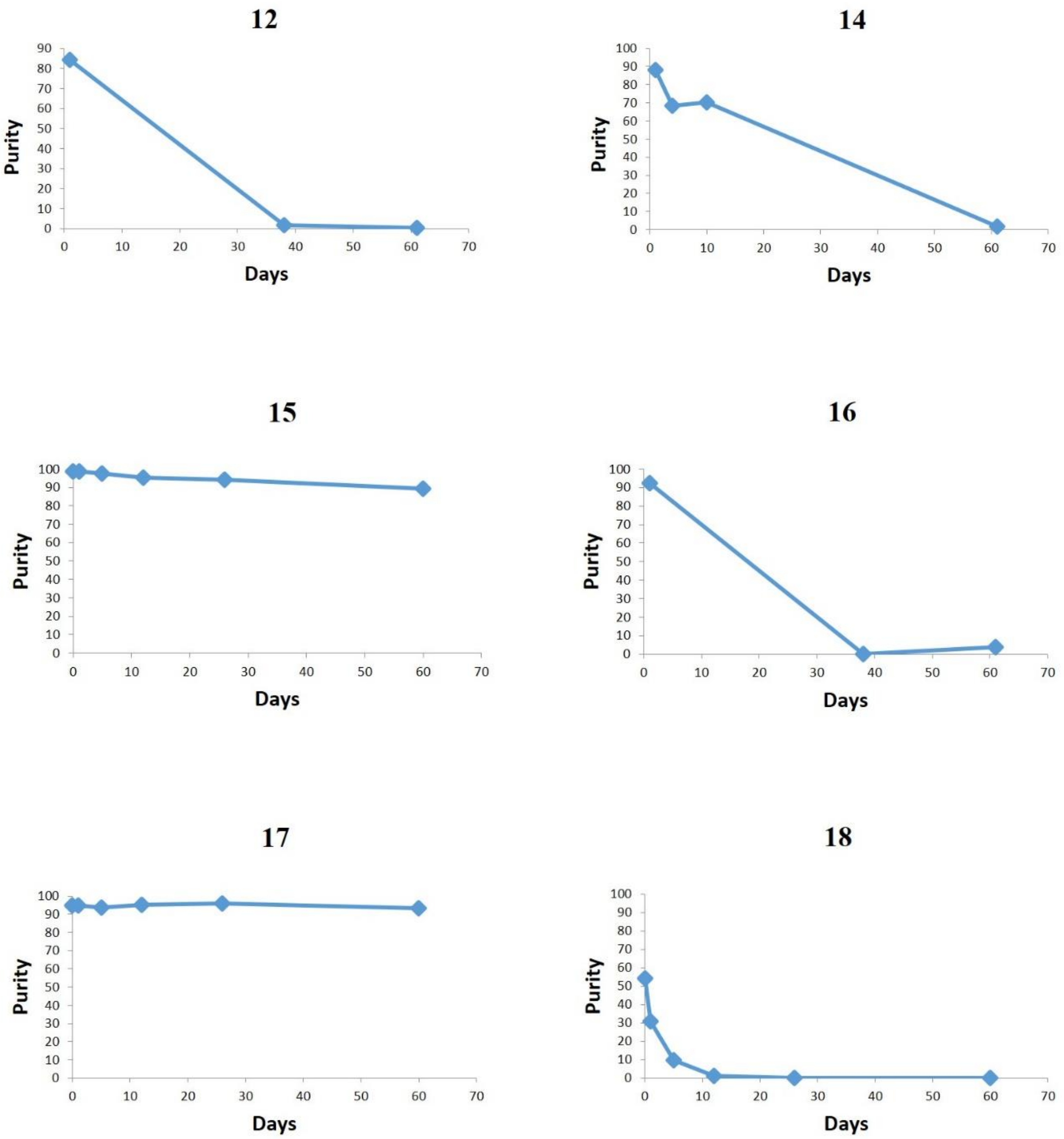

Figure S1. HPLC-MS study of aqueous stability of compounds in aqueous solution with 20% acetonitrile.

3) Antioxidant properties of some natural products.

Table S1. Antioxidant evaluation of several natural products

Comp	Chemical structure	ORAC [a]	ABTS [a]
Resveratrol	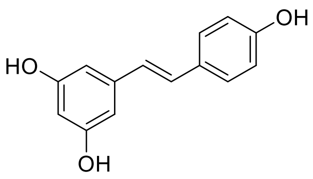	8.08 ± 1.17	2.133 ± 0.018
Quercetin	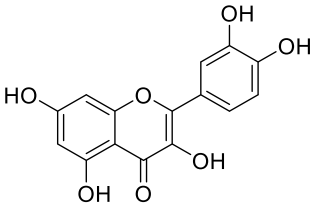	6.61 ± 0.27	5.28 ± 0.29
Hydroxytyrosol	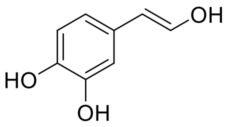	6.33 ± 0.08	0.97 ± 0.05
D-Catechin	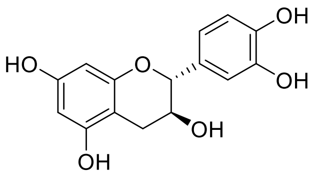	8.59 ± 0.84	3.17 ± 1.22
(-)-Epicatechin	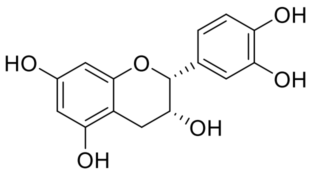	10.03 ± 5.92	5.90 ± 0.73

[a] µmol of trolox / µmol of pure compound.

4) Log P and aqueous solubility of selected compounds.

Table S2. Theoretical Log P values and aqueous thermodynamic solubility measured for selected compounds.

**Comp**	**`Chemical Structure**	**MW**	**LogP Calc**	**Aqueous Solubility [a]**
**3**	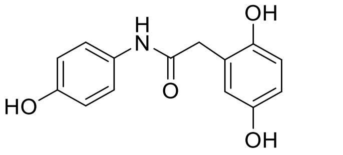	259.26	1.88	1.3 ± 0.1 g/L
**7**	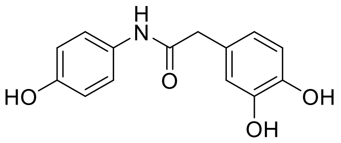	259.26	1.47	3.1 ± 0.1 g/L
**8**	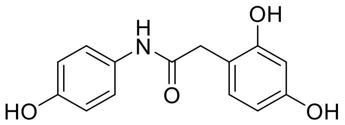	259.26	1.88	4.1 ± 0.1 g/L
**15**	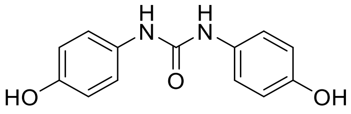	244.25	2.18	0.0626 ± 0.0001 g/L
**17**	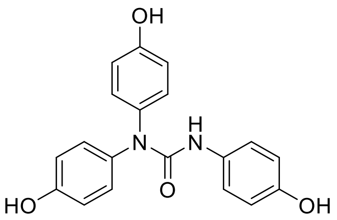	336.35	3.65	0.62 ± 0.01 g/L

[a] Thermodynamic solubility in H_2O (UV).

5) Cell viability assay.

Table S3. IC$_{50}$ values for compounds **3**, **8** and **17**.

**Antioxidant**	**IC$_{50}$ (mM)**
**3**	0.2462
**8**	1.411
**17**	1.789

a) 3

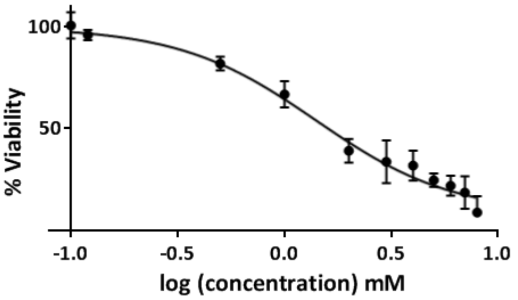

b) 8

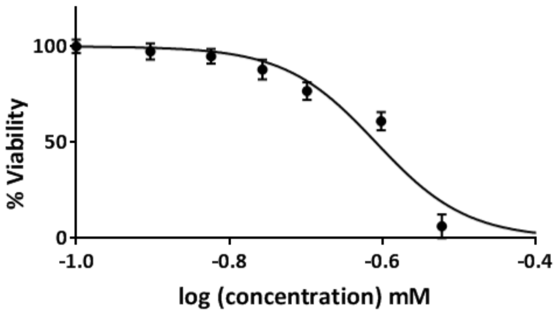

c) 17

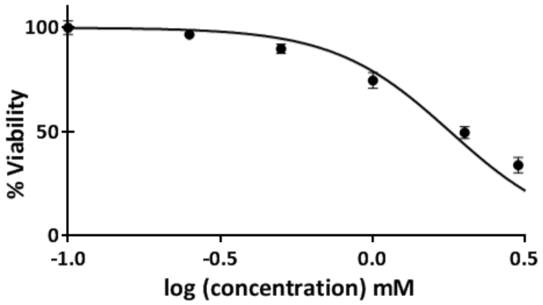

Figure S2. Non-linear fit of %viability vs log (antioxidant concentration) of compounds **3 (**a), **8 (**b) and **17(**c).

7) *In vivo* growth ability induced by selected compounds in yeast

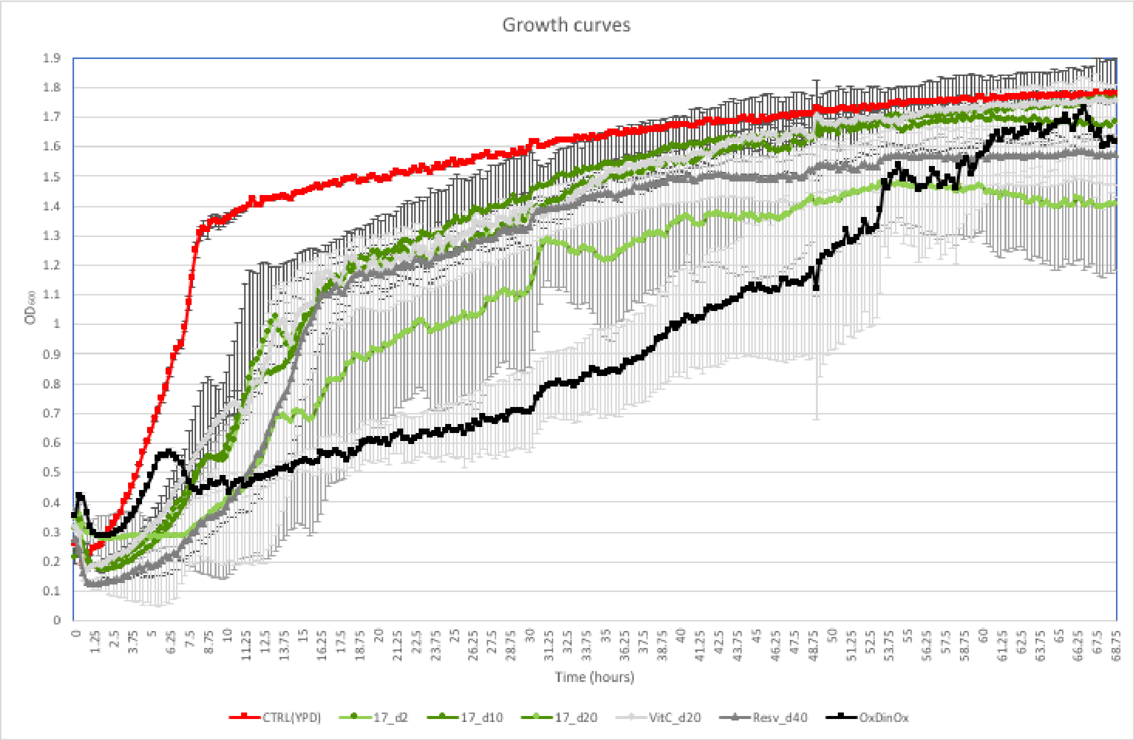

Figure S3. *Saccharomyces cerevisiae* strain BY4741 growth curves (as mean OD595± SEM) vs time). Growth monitorization was performed in a Bioscreen c plate reader and incubator (Oy Growth curves Ab Ldt, Finland), which conducted OD595 (brown filter) measurement each 15 minutes in 100-wells honeycomb plates. Yeasts were recovered from glycerol stocks in YPD for 24h, afterwards, new cultures were established in YPOxD medium to induce oxidative stress for 16h. Stressed yeast cells, were then transferred to new fresh medium in honeycomb plates, where treated with compounds **8**, **15** and **17** at four different doses (2, 10, 20 or 40 µg), and the experiments were conducted in triplicate. Plates also contained control wells with clean un-inoculated medium, and other controls as completely stressed yeasts (OxDinOx; yeast subjected to 16h of oxidative stress and challenged to YPOxD medium for extra 60 hours; 10 samples; in black), non-stressed yeasts (CTRL(YPD), yeast cells grown in YPD medium not subjected to any kind of stress nor supplemented with antioxidant compounds; in red) to ascertain normal growth curve, and two antioxidants controls (Resveratrol and vitamin C challenged at the same 4 doses as compounds; in grey). Graph shows only compound **17** at three doses (2, 10 and 20 µg; in green) and controls (YPD, vitamin C at 20 µg, Resveratrol at 40 µg, and completely stressed cells, OxDinOx) to facilitate reading of the same.
